# Designing for AI literacy: psychological mechanisms and self-determination

**DOI:** 10.3389/fpsyg.2026.1937845

**Published:** 2026-09-14

**Authors:** Suqian Yu, Yuxi Lin, Chen Chen

**Affiliations:** College of Furnishings and Industrial Design, Nanjing Forestry University, Nanjing, Jiangsu, China

**Keywords:** AI literacy, autonomy, competence, fsQCA, open-ended tasks, PLS-SEM, Self-Determination Theory, thinking-based instruction

## Abstract

**Introduction:**

This study examined how instructional approach (skills-based instruction [SBI] versus thinking-based instruction [TBI]) and task type (structured tasks [STT] versus open-ended tasks [OET]) influence university students’ AI literacy performance through autonomy, competence, and relatedness.

**Methods:**

A randomized 2 × 2 between-subjects experiment was conducted with 320 university students assigned equally to four conditions. Participants received video-based instruction and subsequently completed an AIGC image-generation task. AI literacy performance was assessed using an objective task rubric, while the three basic psychological needs were measured using validated self-report scales.

**Results:**

Two-way ANOVA showed significant main effects of instructional approach and task type and a significant interaction effect. TBI produced higher performance than SBI under both task conditions, with a larger advantage under OET. In the structural model, TBI positively predicted autonomy, competence, relatedness, and AI literacy performance. OET positively predicted autonomy, relatedness, and performance but did not significantly predict competence. Competence was the strongest psychological predictor of performance, followed by autonomy, whereas relatedness had no independent effect. Bootstrap analysis showed that instructional approach operated indirectly through autonomy and competence, while task type operated primarily through autonomy. The single factorial interaction term did not retain a significant direct effect after the psychological needs were included. fsQCA further identified multiple sufficient configurations leading to high AI literacy performance.

**Discussion:**

These findings demonstrate that instructional design affects AI literacy through differentiated motivational pathways rather than through a uniform activation of all three psychological needs. Within this single-institution, short-duration experiment, the findings should be interpreted cautiously and require longer, multi-site replication.

## 1 Introduction

The rapid advancement of artificial intelligence-generated content (AIGC) technology is reshaping creative content production and dissemination (Wang et al., 2024). The emergence of user-friendly tools like Midjourney and GPT has lowered technical barriers, making them vital aids for university students (Derevyanko and Zalevska, 2023). However, this trend raises a critical question: as students widely access these tools, is the cultivation of their AI literacy keeping pace (Kasneci et al., 2023)?

AI literacy is an emerging 21st-century competency focusing not merely on operational skills but on a profound understanding of technological principles and ethical implications (Long and Magerko, 2020). Currently, a disconnect is often observed: high-frequency usage of AIGC tools coexists with low levels of AI literacy. Most students remain at basic tool operation, lacking systematic knowledge and critical thinking regarding underlying mechanisms, algorithmic limitations, and ethical risks (Casal-Otero et al., 2023). This gap risks undermining autonomous creative abilities and poses threats to academic integrity and data privacy (Ng et al., 2021a). Consequently, AI literacy must encompass operational skills, understanding of technical principles, critical evaluation ability, and ethical responsibility (Ng et al., 2021b).

Existing research on AIGC in education predominantly relies on the Technology Acceptance Model, focusing on external factors like perceived usefulness (Liu and Ma, 2024; Strzelecki, 2024a,b). While valuable, this work tends to simplify usage behavior as a utilitarian process, failing to fully unveil the intrinsic psychological mechanisms driving deep engagement. Self-Determination Theory posits that sustained behavioral engagement depends on satisfying basic psychological needs: autonomy, competence, and relatedness (Ryan and Deci, 2002). Although recent studies have begun exploring intrinsic motivation in generative AI contexts, empirical research systematically applying this framework to the creative learning context of AIGC image generation remains scarce (Annamalai et al., 2025).

To address these gaps, the present study integrates Self-Determination Theory with a randomized 2 × 2 experimental design comparing skills-based and thinking-based instruction under structured and open-ended task conditions. Objective AI literacy performance serves as the primary learning outcome, while autonomy, competence, and relatedness are examined as potential psychological mechanisms. AI literacy self-awareness is included as a complementary subjective measure rather than as a substitute for demonstrated task performance. Three complementary analytical perspectives are employed: factorial ANOVA estimates the average causal effects of the experimental conditions, structural modeling examines direct and indirect psychological pathways, and fsQCA identifies alternative sufficient configurations associated with high AI literacy performance. The study therefore investigates:

- How instructional approach and task type influence students’ AI literacy performance.
- Whether autonomy, competence, and relatedness mediate the relationship between instructional interventions and learning outcomes.
- Whether multiple configuration pathways can lead to high AI literacy.

The findings are expected to provide a theoretical basis and practical guidance for enhancing students’ AI literacy in AIGC-enabled environments.

## 2 Literature review and theoretical foundation

### 2.1 AI literacy: performance and self-awareness

AI literacy is regarded as a core extension of digital literacy (Chiu et al., 2024). Early research often equated it with operating AI tools, but the definition has evolved toward a multidimensional competency framework (Sperling et al., 2024). Representative frameworks include dimensions like conceptual understanding, critical evaluation, practical application, and ethical awareness (Ng et al., 2022). Genuine AI literacy requires users to collaborate and communicate with AI critically (Long and Magerko, 2020).

In educational contexts, if students only master tool operation but lack deep understanding and critical reflection, they will be ill-equipped to handle potential risks like academic misconduct and algorithmic bias (Southworth et al., 2023). Consequently, the present study assesses AI literacy primarily through demonstrated task performance, supplemented by self-awareness measures of conceptual understanding and critical evaluation.

In the present study, AI literacy performance and AI literacy self-awareness are treated as related but non-equivalent constructs. AI literacy performance refers to competence demonstrated through an authentic AIGC task, including prompt construction, iterative optimization, critical justification, and ethical consideration. AI literacy self-awareness refers to participants’ perceived conceptual understanding and critical evaluation ability. Because perceived competence may not fully correspond to enacted performance, the task-based score is specified as the primary outcome. The self-awareness dimensions are used as complementary indicators to examine whether objective performance is meaningfully associated with participants’ perceived understanding and critical awareness.

### 2.2 Instructional approach: SBI and TBI

In the present study, skills-based instruction (SBI) provided procedural scaffolding through concrete operations, prompt structures, parameter selection, and iterative adjustment strategies. Consistent with cognitive load theory, this structured guidance was intended to help novice learners manage task demands (Sweller et al., 1998) and support their sense of control over tool use. Thinking-based instruction (TBI) places greater emphasis on the principles, limitations, social implications, and ethical consequences of AI systems (Tiruneh et al., 2018). By encouraging learners to interpret, evaluate, and justify their decisions, TBI may support both autonomy and competence: autonomy through greater ownership of judgment and competence through a deeper conceptual basis for solving unfamiliar problems.

These approaches therefore differ primarily in instructional emphasis rather than in whether they support learning at all. SBI emphasizes procedural fluency, whereas TBI emphasizes informed judgment and transfer. Their effects may also depend on whether the accompanying task primarily requires compliance with specified criteria or autonomous interpretation of an ill-structured problem.

### 2.3 Task design: STT and OET

Structured tasks (STT) define explicit goals, required elements, and evaluation criteria (Merrill, 2012). This clarity can reduce ambiguity and cognitive demands, although it may restrict opportunities for autonomous interpretation (Sweller et al., 2019). Open-ended tasks (OET) allow learners to define the meaning, content, and representational form of their responses. Such openness is expected to support autonomy and deeper engagement (Ma, 2009) but may produce competing effects on competence: greater choice may enhance perceived ownership, whereas uncertainty may make task mastery more difficult.

The effects of task openness are therefore unlikely to be uniformly positive or negative across all psychological needs. In accordance with constructive alignment (Biggs, 1996; Biggs et al., 2022), TBI may be particularly compatible with open-ended activities because both require interpretation, evaluation, and higher-order judgment. This alignment is expected to generate a stronger performance advantage than either design feature operating in isolation. Recent instructional models similarly emphasize coordinated rather than isolated design decisions; for example, the CAFE-AI framework integrates content, activity, facilitation, and evaluation across AI-directed, AI-supported, and AI-empowered learning (Suartama et al., 2026).

### 2.4 Self-Determination Theory and basic psychological needs

Self-Determination Theory (SDT) posits that autonomy, competence, and relatedness are basic psychological needs that support intrinsic motivation and deeper engagement (Adams et al., 2017; Ryan and Patrick, 2009). In an AIGC task, autonomy reflects the extent to which learners experience volition and ownership over their creative decisions. Competence reflects perceived effectiveness in generating, evaluating, and refining AI outputs. Relatedness reflects the perceived connection between AI-supported activity and the learner’s wider classroom or learning community.

These needs may make distinct contributions to performance. Autonomy can facilitate exploration and self-directed decision making, competence can support persistence and strategic adjustment, and relatedness can encourage sharing and collaborative meaning making. However, because the present activity was completed individually, the independent effect of relatedness may be less immediate than the effects of autonomy and competence.

### 2.5 Research gaps and hypotheses

Three gaps motivate the present study. First, much of the literature on generative AI in education has emphasized acceptance and intended use rather than demonstrated AI literacy performance. Second, limited experimental evidence is available concerning how instructional emphasis and task openness jointly influence learning. Third, average net-effect analyses cannot determine whether instructional effects operate through distinct psychological pathways or whether multiple combinations of conditions are sufficient for high performance. The present study therefore integrates factorial experimentation, psychological-path analysis, and configurational analysis within a single design.

To address these gaps, this study employs a 2 (instructional approach: skills-based vs. thinking-based) × 2 (task type: structured vs. open-ended) between-subjects design. Figure 1 summarizes the theoretical relationships, and the following hypotheses are proposed:

H1: Thinking-based instruction produces higher AI literacy performance than skills-based instruction.

H2: Open-ended tasks produce higher AI literacy performance than structured tasks.

H3: Instructional approach and task type interact in predicting AI literacy performance, such that the advantage of thinking-based instruction is greater under open-ended tasks.

H4, H5, H6: Autonomy, competence, and relatedness mediate the effect of instructional approach on AI literacy performance, respectively.

H7, H8, H9: Autonomy, competence, and relatedness mediate the effect of task type on AI literacy performance, respectively.

**FIGURE 1:**
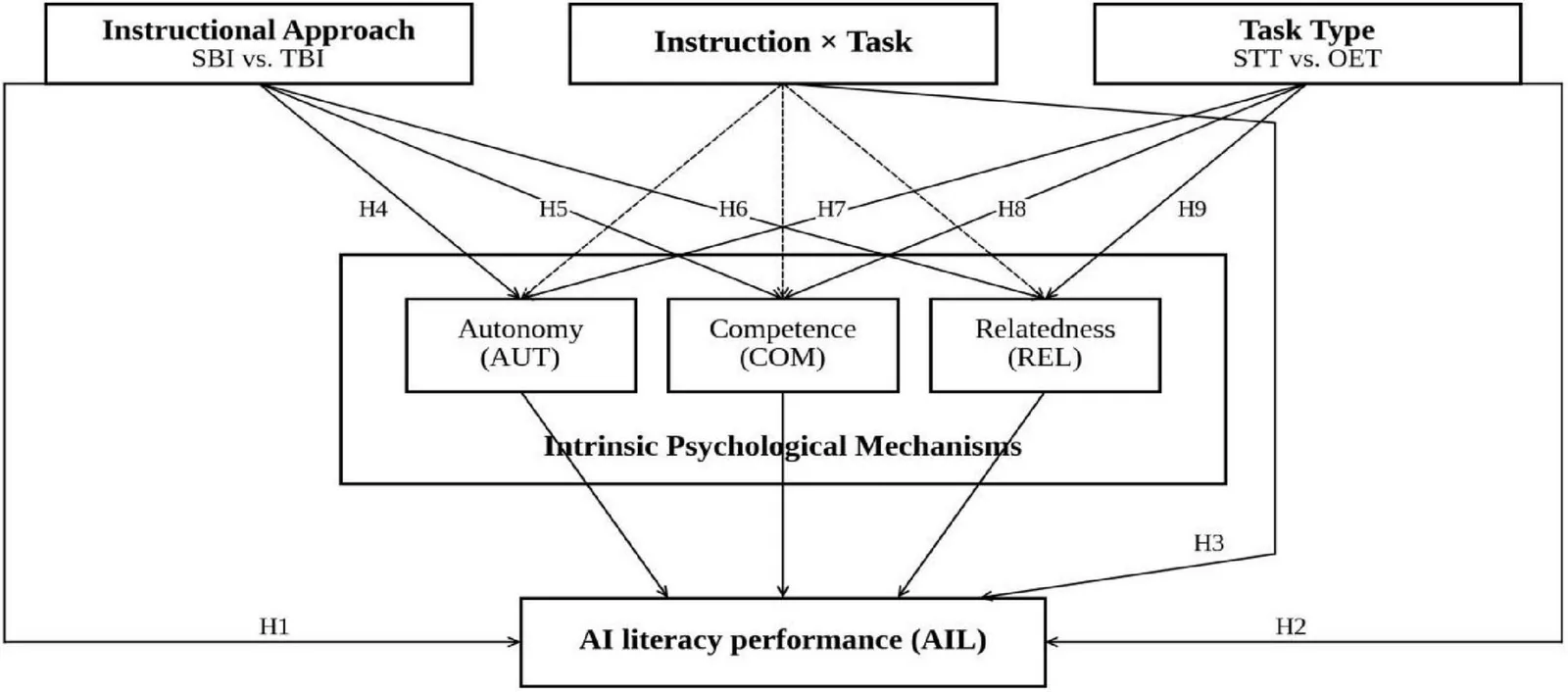
Theoretical model.

Guided by SDT, this research aims to elucidate the psychological mechanisms linking instructional interventions to AI literacy. It examines whether different instructional-task combinations differentially influence objectively assessed performance by stimulating autonomy, competence, and relatedness. This design tests causal effects and explains their underlying pathways, providing theoretical and practical guidance for cultivating advanced AI literacy.

## 3 Research methods

### 3.1 Experimental design and variable coding

This study employed a 2 (instructional approach: skills-based instruction vs. thinking-based instruction) × 2 (task type: structured task vs. open-ended task) between-subjects experimental design. Participants were randomly and equally assigned to one of four experimental groups. A manipulation check was administered immediately after the instructional manipulation. For the structural analysis, instructional approach was coded as 0 = SBI and 1 = TBI, whereas task type was coded as 0 = STT and 1 = OET. Both variables were mean-centered, and a single factorial Instruction × Task interaction term was calculated from the centered predictors. The resulting coding scheme is presented in Table 1.

**TABLE 1:** Experimental condition coding for structural analysis.

Condition	Instruction	Task	Instruction × task
SBI-STT	−0.5	−0.5	0.25
SBI-OET	−0.5	+0.5	−0.25
TBI-STT	+0.5	−0.5	−0.25
TBI-OET	+0.5	+0.5	0.25

### 3.2 Participants

A total of 320 undergraduates (mean age = 20.1, SD = 1.3; 48% male, 52% female) from one university participated after providing informed consent. All had basic experience with AIGC image-generation tools (e.g., Dreamina, Midjourney) and reported regular usage. As shown in Table 2, gender and academic-year distribution were balanced. Participants were randomly and equally assigned to the four experimental groups (*n* = 80 each). An a-priori power analysis (G*Power; Faul et al., 2007) indicated that 128 participants would be sufficient to detect a medium effect [f = 0.25, α = 0.05, power (1−β) = 0.80]; thus, the final sample (*N* = 320) was adequately powered.

**TABLE 2:** Participant demographic characteristics.

Item	Indicators	Frequency	Percent
Gender	Male	153	47.8%
	Female	167	52.2%
Age	18–19	128	40.0%
	20–21	160	50.0%
	22–23	32	10.0%
Academic year	Freshman	92	28.8%
	Sophomore	120	37.5%
	Junior	80	25.0%
	Senior	28	8.7%
AI image-generating software usage frequency	Daily	29	9.1%
	Weekly	207	64.7%
	Monthly	84	26.2%

Prior experience with AIGC image-generation tools was recorded as usage frequency before the experiment. As reported in Table 2, 9.1% of participants used such tools daily, 64.7% weekly, and 26.2% monthly. The distribution of usage frequency did not differ significantly across the four experimental conditions, χ^2^(6) = 9.92, *p* = 0.128, indicating that random assignment produced comparable levels of prior tool exposure. A sensitivity analysis controlling for usage frequency produced the same substantive pattern for instructional approach, task type, and their interaction, and usage frequency itself did not significantly predict AI literacy performance. More detailed baseline proficiency measures should nevertheless be included in future studies.

### 3.3 Experimental materials and manipulation

#### 3.3.1 Instructional materials

Two standardized instructional videos were developed for the experiment: the SBI video lasted 6 min 28 s and the TBI video 6 min 30 s. Both original interventions were delivered in Mandarin Chinese with Simplified Chinese subtitles at 1920 × 1080 resolution by the same Mandarin-speaking narrator. Audio consisted of voice-over only, with no background music or sound effects. Playback was fixed at 1.0×; fast-forwarding, speed adjustment, pausing during the initial presentation, and replay after completion were disabled. Both videos were presented full-screen, required 100% viewing, and the platform recorded start time, viewing progress, completion status, and page-exposure duration.

- A1. The skills-based instruction (SBI) video, Mastering AIGC Prompting: A Guide to Core Techniques, emphasized concrete and observable procedures. Its seven-part progression covered learning objectives, basic prompt structure, subject and detail description, style and generation controls, exclusionary guidance and quality control, iterative optimization, and an integrated example and checklist.
- A2. The thinking-based instruction (TBI) video, A Collaborative Dance with AI: Understanding and Critically Using Image Generation Tools, emphasized explicit reasoning, evaluation, and reflective judgment. Its seven-part progression covered learning objectives, how AIGC generates images, limits of image-generation systems, critical evaluation and verification, bias and stereotypes, copyright/privacy/responsibility, and human-AI collaboration and reflection.

The two videos differed in instructional emphasis while using the same delivery controls. Full English translations of the original Mandarin narration, chapter-level timelines, and chapter-specific instructional screens are provided in Supplementary Sections 1, 2 for peer review and replication. A side-by-side comparison of intervention delivery characteristics is provided in Supplementary Table 1.

To check the effectiveness of the instructional manipulation, a manipulation check was administered immediately after participants received the instructional materials. The manipulation check comprised two items, both measured on a 7-point Likert scale (1 = “Strongly Disagree,” 7 = “Strongly Agree”):

- Item I (targeting thinking-based instruction): “I believe the instructional content just received placed greater emphasis on inspiring my critical thinking.”
- Item II (targeting skills-based instruction): “I believe the instructional content just received placed greater emphasis on teaching specific usage techniques and procedures.”

The TBI group scored significantly higher on “inspiring critical thinking” (M = 5.82, SD = 0.91) than the SBI group (M = 3.45, SD = 1.12), *t*(318) = 20.76, *p* < 0.001. Conversely, the SBI group scored significantly higher on “teaching specific techniques” (M = 5.95, SD = 0.87) than the TBI group (M = 3.61, SD = 1.04), *t*(318) = 21.83, *p* < 0.001, confirming the intended differentiation between the instructional conditions.

#### 3.3.2 Task type materials

All groups used the same AIGC tool (Dreamina) to complete an image-generation task, while task type was manipulated by varying the degree of constraint imposed on the generation goal.

- B1. Structured task: The instructions required participants to generate an image that must include “buildings with solar panels, wind-power facilities, a clear river, a sunny and bright atmosphere, and a photorealistic style.” This task had explicit objectives and constrained creative freedom.
- B2. Open-ended task: The instructions asked participants to create an image that expresses the concept of “a future of harmonious coexistence between humans and nature” and encouraged them to fully utilize their imagination. This task emphasized free interpretation and conceptual expression.

To ensure comparability of the evidence available for performance scoring, participants in both task conditions submitted the same core materials: the final generated image, the final prompt, an image-generation process statement documenting the major revisions made during generation, and a final-output selection rationale. The submission format was identical across the two task conditions; task type differed only in the degree of constraint imposed on the generation goal. Full original Mandarin instructions, English translations, the common task-introduction screen, and the standardized submission form are provided in Supplementary Section 3.

All participants used the web-based version of Dreamina during data collection. The platform displayed the image-generation model as Image 5.0 lite. All participants used the same standardized account configuration and fixed generation settings: a 3:4 aspect ratio, 768 × 1024 px output resolution, four candidate images per generation request, and a maximum of five generation requests (up to 20 candidate images). Reference-image upload and automatic prompt enhancement were not allowed; in-platform inpainting/local redrawing was allowed when necessary, whereas other post-generation image editing was not allowed. Detailed platform settings, experimental restrictions, and the data-collection period are reported in Supplementary Table 3.

### 3.4 Measures

#### 3.4.1 AI literacy performance

An objective assessment was conducted using the “AIGC Practical Task Scoring Rubric” developed for this study, applied to the submitted task materials (final prompts, image-generation process statements, and final-output selection rationales). The final generated image was retained as supporting task evidence but was not assigned a separate rubric score. The rubric evaluated demonstrated performance rather than participants’ perceptions of their own ability. The rubric comprised four dimensions:

- Prompt complexity: Ranging from basic description to elaborated and stylized description (Khan, 2024) (1–3 points).
- Iterative optimization process: Ranging from no iteration to strategic iteration (Deshmukh, 2024) (1–3 points).
- Critical selection and justification: Ranging from subjective reasons to comprehensive justifications (Ng et al., 2021b) (1–3 points).
- Ethical consideration: Ranging from not mentioned to in-depth discussion (Long and Magerko, 2020) (1–3 points).

The total score ranged from 4 to 12, with higher scores indicating better AI literacy performance. Two instructor raters who were blind to the experimental conditions independently evaluated all submissions. The intraclass correlation coefficient (ICC) was 0.89, indicating high inter-rater agreement. The rubric dimensions were aligned *a priori* with established conceptualizations of AI literacy concerning practical application, critical evaluation, and responsible use.

Beyond inter-rater reliability, additional validity evidence based on relations to theoretically relevant variables was provided by the positive correlations between objective AI literacy performance and self-reported conceptual understanding (*r* = 0.396, *p* < 0.001) and critical evaluation (*r* = 0.474, *p* < 0.001). These moderate associations indicate that the rubric is meaningfully related to perceived AI literacy while remaining empirically distinct from self-awareness. Because no external criterion or independent validation sample was available, the rubric should be regarded as a study-specific performance measure rather than a fully validated general-purpose AI literacy instrument.

#### 3.4.2 Basic psychological need satisfaction

Autonomy, competence, and relatedness were measured after the task using three-item, seven-point Likert scales ranging from 1 (“strongly disagree”) to 7 (“strongly agree”). The items were adapted from established Self-Determination Theory scales (Ryan et al., 2009). Autonomy captured perceived volition and control over the creative process. Competence captured confidence, proficiency, and perceived ability to improve generated outputs. Relatedness captured perceived connection, communication, and belonging within the learning community. The three constructs were modeled as reflective psychological-need variables. They were specified as the theoretically proposed psychological mediators in the PLS-SEM model and were also included as psychological conditions in the fsQCA.

#### 3.4.3 AI literacy self-awareness

AI literacy self-awareness was assessed through conceptual understanding and critical evaluation. Conceptual understanding captured participants’ perceived knowledge of how AIGC systems, prompts, and model limitations operate. Critical evaluation captured their perceived ability to examine output accuracy, recognize potential bias, and evaluate the strengths and weaknesses of AIGC tools. These two dimensions were included as complementary validation measures. They were not specified as psychological mediators or primary outcomes and were analyzed descriptively and correlationally rather than being included in the PLS-SEM or fsQCA models.

Table 3 reports the measurement properties of the three Self-Determination Theory psychological-need constructs included in the structural and configurational analyses. The square roots of AVE exceeded the inter-construct correlations, supporting discriminant validity (Hilkenmeier et al., 2020), as shown in Table 4. Conceptual understanding and critical evaluation are retained as secondary AI-literacy self-awareness measures; their measurement properties are reported separately in Supplementary Table 2. Reliability was assessed using established psychometric criteria (Nunnally and Bernstein, 1994).

**TABLE 3:** Reliability and convergent validity of the psychological-need measures.

Indicators	Loadings	Cronbach’s alpha	rho_A	Composite reliability	AVE
Autonomy					
I feel I can freely express my ideas when using AIGC tools.	0.891	0.887	0.889	0.922	0.801
I feel that I use this tool of my own volition, rather than being compelled to.	0.902				
I feel that I am in control of the creative process when using AIGC tools.	0.896				
Competence					
I feel confident in using AIGC tools to generate the image results I want.	0.923	0.913	0.917	0.942	0.848
Through practice, I feel I am becoming more proficient in using AIGC tools.	0.912				
When the generated result is unsatisfactory, I know how to adjust the prompts to improve it.	0.928				
Relatedness					
When using AIGC tools for learning, I feel I am part of our learning community (class/group).	0.905	0.891	0.892	0.929	0.812
I am willing to share and discuss the works and experiences I have created using AIGC tools with my classmates.	0.898				
I believe that using AIGC tools can facilitate communication and collaboration with my classmates in learning.	0.901				

Autonomy, competence, and relatedness were included as mediators in the PLS-SEM model and as psychological conditions in the fsQCA. Measurement properties for the secondary conceptual-understanding and critical-evaluation measures are reported separately in Supplementary Table 2.

**TABLE 4:** Discriminant validity of the psychological-need constructs.

Indicators	1	2	3
1. Autonomy	0.895	0.921	0.901
2. Competence	0.452		
3. Relatedness	0.411	0.387	

The table reports the Fornell–Larcker assessment for the three psychological-need constructs included in the structural and configurational models. The diagonal values are square roots of AVE. Discriminant-validity information for conceptual understanding and critical evaluation is reported separately in Supplementary Table 2.

### 3.5 Experimental procedure

After providing informed consent, participants completed a pre-task questionnaire covering demographic characteristics and prior AIGC-tool usage. Participants were assigned by the online experimental platform using computer-generated permuted-block randomization with a 1:1:1:1 allocation ratio, resulting in 80 participants in each of the four experimental conditions. Participants then viewed the instructional video corresponding to their assigned instructional condition under the standardized delivery controls described above and immediately completed the two manipulation-check items. They subsequently received either the structured-task or open-ended-task instructions and entered a 10-min Dreamina image-generation stage conducted under the standardized platform settings and restrictions described in Supplementary Section 3. At the end of the task, participants submitted the final generated image, final prompt, image-generation process statement, and final-output selection rationale using the standardized submission form. Finally, participants completed the measures of autonomy, competence, relatedness, and AI literacy self-awareness. The full experimental session required approximately 20–25 min. A detailed procedural flow is provided in Supplementary Figure 3.3.

### 3.6 Data analysis strategy

Four complementary analytical procedures were employed. First, a two-way factorial ANOVA estimated the main and interaction effects of instructional approach and task type on AI literacy performance, with simple-effect comparisons used to interpret the interaction. Second, descriptive statistics and Pearson correlations examined relationships among objective AI literacy performance, the three psychological needs, and the two AI literacy self-awareness dimensions; the performance–self-awareness correlations also provided additional validity evidence for the task rubric based on relations to theoretically relevant variables. Third, a PLS-SEM structural model examined direct and indirect psychological pathways. The PLS-SEM analysis followed established recommendations for model specification and reporting (Chin, 1998; Hair et al., 2014, 2019). Finally, fsQCA examined causal asymmetry and equifinality by identifying alternative sufficient configurations associated with high AI literacy performance (Kraus et al., 2018).

The mean-centered experimental predictors and their single product interaction term described in Section “3.1 Experimental design and variable coding” were entered into SmartPLS as observed exogenous predictors. Instructional approach, task type, and the interaction term were specified as predictors of autonomy, competence, relatedness, and AI literacy performance; the three psychological needs were further specified as predictors of performance. No cell-specific interaction variables (e.g., TBI × OET, TBI × STT, SBI × OET, or SBI × STT) were specified. Direct and indirect effects were evaluated using 5,000 bootstrap resamples and 95% confidence intervals. Inner VIF values were examined for collinearity, model explanatory power using *R*^2^ and adjusted *R*^2^, and predictive relevance using blindfolding-based Q^2^ with an omission distance of seven.

For the fsQCA analysis, instructional approach and task type were treated as dichotomous crisp experimental conditions, whereas autonomy, competence, relatedness, and AI literacy performance were calibrated using the 95th, 50th, and 5th percentiles as anchors for full membership, crossover, and full non-membership, respectively. Necessity was evaluated using a consistency criterion of 0.90. For the sufficiency analysis, the truth table was constructed using a case-frequency threshold of 5, a consistency threshold of 0.80, and a PRI consistency threshold of 0.70. The intermediate solution was used for substantive interpretation. Core conditions were those appearing in both the parsimonious and intermediate solutions, whereas peripheral conditions appeared only in the intermediate solution.

The inferential approaches address non-equivalent questions. ANOVA estimates average experimental main and interaction effects; PLS-SEM examines how the effects may operate through net psychological pathways while accounting for the measurement model; and fsQCA identifies combinations of conditions sufficient for high performance. Thus, the configurational analysis complements rather than substitutes for the net-effect analyses.

## 4 Results

A total of 320 valid responses were collected for this study. The sample was evenly distributed across the four experimental groups (Skills-Based/Structured, Skills-Based/Open-Ended, Thinking-Based/Structured, Thinking-Based/Open-Ended), with 80 participants in each group.

### 4.1 ANOVA: effects of instructional approach and task type

A two-way ANOVA (Ståhle and Wold, 1989) revealed a significant main effect of instructional approach on AI literacy performance, *F*(1, 316) = 50.79, *p* < 0.001, ηp^2^ = 0.138. Participants receiving TBI (M = 9.21, SD = 1.99) achieved higher performance than those receiving SBI (M = 7.69, SD = 2.05). A significant main effect of task type was also observed, *F*(1, 316) = 31.45, *p* < 0.001, ηp^2^ = 0.091. Participants completing OET (M = 9.05, SD = 1.97) outperformed those completing STT (M = 7.85, SD = 2.18).

The interaction between instructional approach and task type was significant, *F*(1, 316) = 7.22, *p* = 0.008, ηp^2^ = 0.022. Under OET, the TBI group (M = 10.10, SD = 1.52) outperformed the SBI group (M = 8.00, SD = 1.81), *t*(158) = 7.95, *p* < 0.001. Under STT, the TBI group (M = 8.33, SD = 2.01) also outperformed the SBI group (M = 7.38, SD = 2.24), *t*(158) = 2.82, *p* = 0.005. The TBI–SBI difference was therefore present under both task conditions but was larger under OET. The interaction pattern is shown in Figure 2.

**FIGURE 2:**
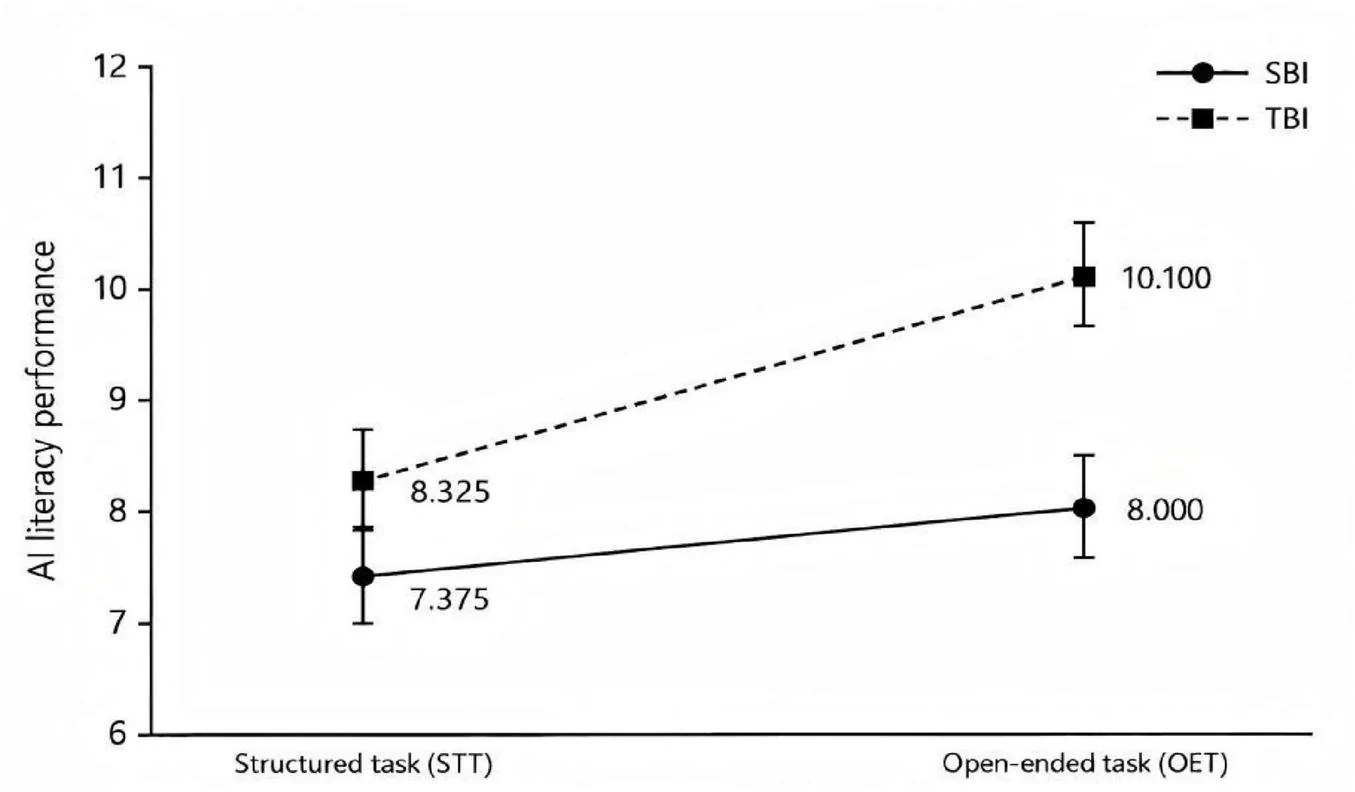
Interaction between instructional approach and task type on AI literacy performance. Error bars represent 95% confidence intervals.

Viewed from the task-type perspective, OET significantly improved performance relative to STT under TBI, *t*(158) = 6.29, *p* < 0.001, whereas the difference under SBI was smaller and did not reach statistical significance, *t*(158) = 1.94, *p* = 0.054. These results support H1, H2, and H3.

### 4.2 Descriptive statistics: preliminary examination of variable relationships

Descriptive statistics and Pearson correlations (Table 5) were examined to characterize relationships among AI literacy performance, self-awareness, and the three basic psychological needs (Nick, 2007). Objective AI literacy performance was positively correlated with conceptual understanding (*r* = 0.396, *p* < 0.001) and critical evaluation (*r* = 0.474, *p* < 0.001). These associations support the conceptual relatedness of performance and self-awareness while indicating that they are not interchangeable.

**TABLE 5:** Descriptive statistics and Pearson correlations.

Indicators	M	SD	1	2	3	4	5
1. Conceptual understanding	5.231	0.894	—	—	—	—	—
2. Critical evaluation	5.443	0.918	0.504				
3. Autonomy	5.049	0.912	0.414	0.398			
4. Competence	5.306	0.950	0.446	0.454	0.591		
5. Relatedness	4.934	0.978	0.376	0.318	0.519	0.407	
6. AI literacy performance	8.450	2.158	0.396	0.474	0.494	0.541	0.368

Off-diagonal values are Pearson correlation coefficients. All reported correlations have *p* < 0.001. Conceptual understanding and critical evaluation were analyzed as complementary self-awareness measures and were not included as mediators or configurational conditions.

AI literacy performance was also positively correlated with autonomy (*r* = 0.494, *p* < 0.001), competence (*r* = 0.541, *p* < 0.001), and relatedness (*r* = 0.368, *p* < 0.001). Competence showed the strongest bivariate association with performance. The psychological-need constructs were moderately intercorrelated, supporting estimation of their independent effects in the subsequent structural model.

### 4.3 PLS-SEM analysis: direct and indirect psychological pathways

#### 4.3.1 Structural path estimates

The structural model included the two centered experimental predictors and a single Instruction × Task product term. Instructional approach, task type, and their interaction predicted autonomy, competence, relatedness, and AI literacy performance; the three psychological needs were simultaneously included as predictors of performance. Table 6 summarizes the standardized structural path estimates. The estimated structural model is shown in Figure 3.

**TABLE 6:** Structural path estimates.

Path	β	Boot. SD	*t*	*p*	95% CI	f^2^	VIF
Instruction → AUT	0.269	0.050	5.398	< 0.001	[0.173, 0.365]	0.083	1.000
Task → AUT	0.221	0.050	4.409	< 0.001	[0.123, 0.318]	0.056	1.000
Instruction × task → AUT	0.083	0.053	1.573	0.116	[−0.020, 0.187]	0.008	1.000
Instruction → COM	0.220	0.052	4.197	< 0.001	[0.111, 0.320]	0.052	1.000
Task → COM	0.086	0.055	1.561	0.119	[−0.023, 0.195]	0.008	1.000
Instruction × task → COM	0.105	0.055	1.905	0.057	[−0.003, 0.214]	0.012	1.000
Instruction → REL	0.153	0.053	2.858	0.004	[0.045, 0.255]	0.025	1.000
Task → REL	0.215	0.051	4.206	< 0.001	[0.116, 0.313]	0.050	1.000
Instruction × task → REL	0.054	0.055	0.992	0.321	[−0.052, 0.161]	0.003	1.000
Instruction → AIL	0.229	0.045	5.091	< 0.001	[0.145, 0.321]	0.085	1.089
Task → AIL	0.204	0.044	4.593	< 0.001	[0.118, 0.292]	0.068	1.077
Instruction × task → AIL	0.081	0.043	1.886	0.059	[−0.004, 0.162]	0.011	1.013
AUT → AIL	0.132	0.061	2.172	0.030	[0.010, 0.249]	0.016	1.890
COM → AIL	0.358	0.058	6.205	< 0.001	[0.241, 0.468]	0.142	1.595
REL → AIL	0.071	0.050	1.428	0.153	[−0.027, 0.166]	0.006	1.423

Instructional approach was coded SBI = 0 and TBI = 1; task type was coded STT = 0 and OET = 1. Both predictors were mean-centered before calculating the single interaction term. AUT, autonomy; COM, competence; REL, relatedness; AIL, AI literacy performance.

**FIGURE 3:**
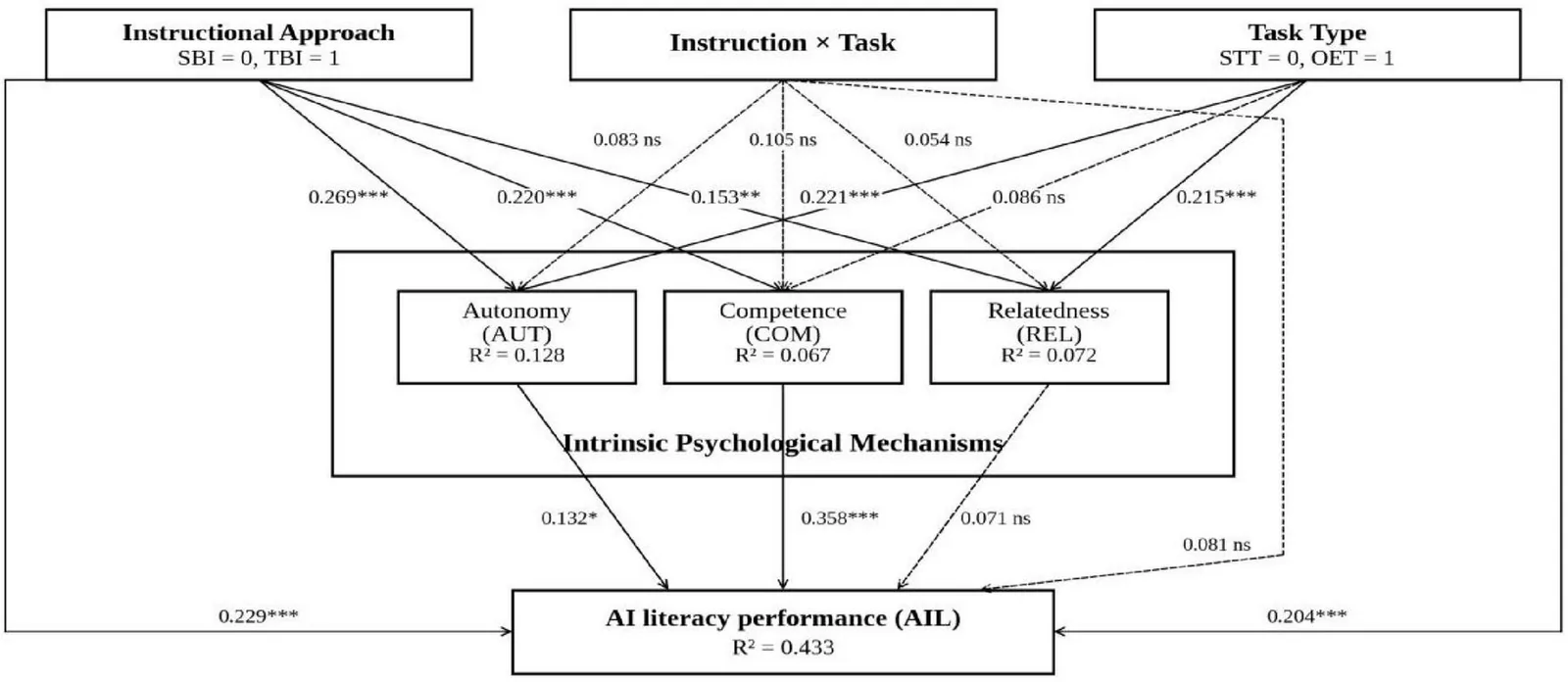
Structural model (***p < 0.001, **p < 0.01, *p < 0.05, NS, not-significant).

Instructional approach had significant positive effects on autonomy (β = 0.269, *p* < 0.001), competence (β = 0.220, *p* < 0.001), and relatedness (β = 0.153, *p* = 0.004). It also retained a significant direct effect on AI literacy performance after the psychological needs were included (β = 0.229, *p* < 0.001).

Task type positively predicted autonomy (β = 0.221, *p* < 0.001) and relatedness (β = 0.215, *p* < 0.001), but its effect on competence was not statistically significant (β = 0.086, *p* = 0.119). Task type retained a significant direct effect on AI literacy performance (β = 0.204, *p* < 0.001).

The single Instruction × Task term did not significantly predict autonomy (β = 0.083, *p* = 0.116), competence (β = 0.105, *p* = 0.057), relatedness (β = 0.054, *p* = 0.321), or AI literacy performance (β = 0.081, *p* = 0.059) after the psychological needs were included. This nonsignificant structural interaction does not contradict the significant ANOVA interaction: ANOVA estimates the overall performance-level interaction, whereas the structural path represents the remaining direct interaction after autonomy, competence, and relatedness are controlled.

Among the psychological needs, competence was the strongest independent predictor of AI literacy performance (β = 0.358, *p* < 0.001), followed by autonomy (β = 0.132, *p* = 0.030). Relatedness did not have a significant independent effect (β = 0.071, *p* = 0.153).

#### 4.3.2 Mediation analysis

Specific indirect effects were evaluated using the same 5,000 bootstrap resamples and 95% confidence intervals reported in Table 7.

**TABLE 7:** Specific indirect and total effects.

Predictor	Path	β	Boot. mean	Boot. SD	*p*	95% CI
Instruction	Instruction → AUT → AIL	0.035	0.035	0.018	0.034	[0.003, 0.073]
	Instruction → COM → AIL	0.079	0.078	0.023	< 0.001	[0.037, 0.125]
	Instruction → REL → AIL	0.011	0.011	0.009	0.157	[−0.004, 0.030]
	Total indirect effect	0.125	0.124	0.028	< 0.001	[0.070, 0.178]
	Total effect	0.354	0.354	0.048	< 0.001	[0.258, 0.445]
Task	Task → AUT → AIL	0.029	0.029	0.015	0.034	[0.002, 0.062]
	Task → COM → AIL	0.031	0.031	0.020	0.121	[−0.008, 0.073]
	Task → REL → AIL	0.015	0.015	0.012	0.150	[−0.006, 0.041]
	Total indirect effect	0.075	0.075	0.028	0.008	[0.021, 0.128]
	Total effect	0.279	0.280	0.048	< 0.001	[0.186, 0.373]

Instructional approach had significant indirect effects on AI literacy performance through autonomy (β = 0.035, 95% CI [0.003, 0.073]) and competence (β = 0.079, 95% CI [0.037, 0.125]). The indirect effect through relatedness was not significant (β = 0.011, 95% CI [−0.004, 0.030]). The total indirect effect of instructional approach was significant (β = 0.125, 95% CI [0.070, 0.178]), as was its total effect (β = 0.354, 95% CI [0.258, 0.445]). Because the direct effect remained significant, autonomy and competence partially mediated the effect of instructional approach. H4 and H5 were supported, whereas H6 was not supported.

Task type had a significant indirect effect through autonomy (β = 0.029, 95% CI [0.002, 0.062]). The indirect effects through competence (β = 0.031, 95% CI [−0.008, 0.073]) and relatedness (β = 0.015, 95% CI [−0.006, 0.041]) were not significant. The total indirect effect of task type was significant (β = 0.075, 95% CI [0.021, 0.128]), as was its total effect (β = 0.279, 95% CI [0.186, 0.373]). Because the direct effect remained significant, autonomy provided partial mediation. H7 was supported, whereas H8 and H9 were not supported.

Overall, instructional approach influenced performance indirectly through both autonomy and competence, whereas the indirect effect of task openness was concentrated primarily in autonomy.

The mediation results therefore indicate differentiated rather than uniform psychological pathways.

#### 4.3.3 Explanatory power, predictive relevance, and collinearity

The model explained 12.8% of the variance in autonomy (*R*^2^ = 0.128), 6.7% in competence (*R*^2^ = 0.067), 7.2% in relatedness (*R*^2^ = 0.072), and 43.3% in AI literacy performance (*R*^2^ = 0.433). The corresponding adjusted *R*^2^ values were 0.120, 0.058, 0.063, and 0.423, respectively. Q^2^ values were greater than zero for all endogenous constructs: autonomy = 0.089, competence = 0.031, relatedness = 0.040, and AI literacy performance = 0.401, indicating predictive relevance, although it was limited for competence and relatedness. Inner VIF values ranged from 1.000 to 1.890, providing no indication of problematic multicollinearity.

### 4.4 fsQCA—multiple pathways to high AI literacy

fsQCA was used to complement the average net-effect analyses by examining whether distinct combinations of instructional approach, task type, autonomy, competence, and relatedness were sufficient for high AI literacy performance. Instructional approach was represented by membership in TBI, with non-membership representing SBI. Task type was represented by membership in OET, with non-membership representing STT. The psychological variables and outcome were calibrated using the 95th, 50th, and 5th percentiles as the full-membership, crossover, and full-non-membership anchors.

No single condition or its negation reached the consistency threshold for necessity. For the sufficiency analysis, the truth-table analysis identified three configurations that met the predefined frequency, consistency, and PRI consistency criteria. The three sufficient configurations are presented in Table 8. Their consistencies were 0.923, 0.896, and 0.941; raw coverages were 0.386, 0.357, and 0.458; and unique coverages were 0.089, 0.072, and 0.156. Overall solution coverage was 0.588 and overall solution consistency was 0.913, indicating that the configurations jointly covered a substantial proportion of high-performance cases with high consistency.

**TABLE 8:** fsQCA sufficient configurations.

Variable	1	2	3
TBI	•		•
SBI		•	
OET		•	•
STT	•		
COM	•	⋅	•
AUT	⋅	•	⋅
REL		⋅	
Raw coverage	0.386	0.357	0.458
Unique coverage	0.089	0.072	0.156
Consistency	0.923	0.896	0.941
Solution coverage	0.588		
Solution consistency	0.913		

“•” indicates a core condition, “⋅” indicates a peripheral condition, and a blank cell indicates that the condition is irrelevant to the configuration.

Configuration 1 combined TBI, the absence of OET (i.e., STT), and high competence as core conditions, with autonomy and relatedness as peripheral conditions. Configuration 2 combined the absence of TBI (i.e., SBI), OET, and high autonomy as core conditions, with competence and relatedness as peripheral conditions. Configuration 3 combined TBI, OET, and high competence as core conditions, with autonomy as a peripheral condition and relatedness irrelevant. Configuration 3 had the largest raw coverage, although all three configurations met the consistency requirement for sufficient conditions.

These results demonstrate equifinality: high AI literacy performance can arise from multiple combinations rather than from a single universally optimal instructional design.

## 5 Discussion

### 5.1 Summary of key findings

Three principal findings emerged. First, TBI and OET both improved objective AI literacy performance. TBI outperformed SBI under both task conditions, but the difference was substantially larger under OET. OET produced a pronounced performance benefit under TBI, whereas its benefit under SBI was comparatively small.

Second, the psychological mechanisms were differentiated rather than uniform. TBI positively predicted all three psychological needs, but its indirect relationship with performance operated through autonomy and competence. OET positively predicted autonomy and relatedness, but only autonomy transmitted its effect to performance. Competence was the strongest independent psychological predictor of AI literacy performance, followed by autonomy, whereas relatedness did not have an independent net effect.

Third, the configurational analysis identified multiple sufficient pathways to high performance. The fsQCA results therefore complemented the net-effect model by showing that a condition without a significant independent linear effect may still contribute within a specific combination of instructional and psychological conditions.

### 5.2 Conditional effects of instructional approach and task design

The factorial results demonstrate that the effectiveness of instructional approach depends partly on task design. TBI produced higher performance under both task types, indicating that conceptual and critical instruction was beneficial even when the task was structured. However, the larger TBI–SBI difference under OET suggests that open-ended activities provide greater opportunities for learners to apply conceptual understanding, critical evaluation, and autonomous judgment.

The significant ANOVA interaction should be interpreted as an overall performance-level effect. The interaction term did not retain a statistically significant direct effect in the structural model after autonomy, competence, and relatedness were included. Thus, the results support conditional amplification of TBI at the performance level but do not establish a direct synergistic effect on each psychological need.

This distinction avoids treating statistical interaction and psychological mediation as interchangeable. Instruction–task alignment may affect performance through several small or distributed processes that are not captured by one individual mediator.

### 5.3 Differentiated roles of autonomy, competence, and relatedness

The structural results provide differentiated support for SDT. Competence was the strongest independent predictor of AI literacy performance, indicating that learners’ perceived ability to generate, evaluate, and improve AI outputs was central to successful task performance. Autonomy also made a significant contribution, consistent with the importance of volition and ownership in exploratory AI-supported learning.

TBI influenced performance indirectly through both autonomy and competence. Conceptual and critical instruction may increase autonomy by providing learners with a basis for independent judgment rather than requiring mechanical compliance with procedures. It may support competence by enabling learners to diagnose unfamiliar outputs and select appropriate corrective strategies.

Task openness operated indirectly primarily through autonomy. Greater choice and interpretive freedom appear to benefit performance when learners experience genuine ownership over the task. By contrast, task type did not significantly influence competence after the other predictors were considered, providing no evidence that OET either systematically undermined or enhanced competence.

Relatedness was positively influenced by both instructional approach and task openness but did not independently predict objective performance. This result may reflect the individually completed nature of the task. Relatedness may be more consequential for collaborative participation, persistence, or classroom engagement than for immediate individual performance. It may also operate configurationally rather than as an independent net-effect predictor.

### 5.4 Multiple configurational pathways

The fsQCA results extend the structural findings by demonstrating equifinality. High performance was associated with a TBI–structured-task configuration centered on competence, an SBI–open-ended-task configuration centered on autonomy, and a TBI–open-ended-task configuration centered on competence. These patterns indicate that instructional limitations can be compensated for by favorable psychological conditions.

The configurational and structural findings address different causal questions. The structural model estimates the average independent contribution of each condition while holding other variables constant. fsQCA identifies combinations that are sufficient for high performance. Consequently, the absence of an independent relatedness effect does not imply that relatedness is irrelevant in every learning configuration.

Configuration 3 had the largest raw coverage, but it should not be described as a universally “best” model. Its relevance is conditional on the present sample, task, tool, and calibration decisions.

### 5.5 Practical implications

The findings suggest three instructional principles. First, AI literacy instruction should move beyond procedural prompting and include model principles, limitations, critical evaluation, and ethical responsibility. Second, open-ended tasks are most useful when learners receive sufficient conceptual support to make and justify independent decisions. Third, instructional support should prioritize competence and autonomy through worked examples, diagnostic feedback, opportunities for revision, and meaningful choice.

Relatedness may require more explicit collaborative design than was present in the current individual task. Peer critique, shared prompt repositories, collaborative evaluation, and group reflection may provide stronger opportunities for relatedness to influence learning outcomes.

### 5.6 Limitations and future research

Several alternative explanations should be considered. Although the submission format and core evidence were standardized across the two task conditions, the open-ended task afforded greater interpretive freedom, and the rubric rewarded prompt complexity, iterative optimization, justification, and ethical reflection. Higher scores may therefore partly reflect alignment between the affordances of open-ended task design and the assessment criteria rather than a general advantage of open-ended pedagogy. The brief intervention may also have produced novelty or demand effects, and prior AIGC experience may have helped some participants translate the instruction into prompts more efficiently.

Evidence from other designs suggests that the optimal degree of openness depends on learner expertise, duration, and scaffolding. Cognitive load theory predicts benefits of explicit structure for novices when task complexity is high (Sweller et al., 2019). In addition, a 16-week evaluation of the structured CAFE-AI model reported gains in AI literacy and growth mindset relative to conventional e-learning (Suartama et al., 2026). These findings do not negate the present interaction effect; they indicate that open-ended tasks should not be treated as universally superior. A more defensible implication is to align task openness with instructional support and learners’ prior experience.

Several limitations qualify the interpretation of the findings. First, participants were recruited from a single university, and all had prior experience with AIGC image-generation tools. The findings may therefore not generalize to students from other institutions, disciplines, educational levels, or complete beginners.

Second, the intervention was brief and the outcomes were measured immediately after a single 20–25-min activity. The study does not establish whether the observed effects persist over time or transfer to different AI tools, content domains, or authentic academic tasks. Longitudinal studies should examine delayed retention, transfer, and sustained responsible use.

Third, autonomy, competence, and relatedness were measured after task completion. Successful task performance may itself have increased perceived autonomy or competence. The indirect effects are therefore consistent with the proposed psychological mechanisms but do not establish their temporal ordering as conclusively as repeated or longitudinal mediation designs would.

Fourth, instructional content and assessment dimensions were partly aligned. The SBI video directly addressed prompt construction and iteration, whereas the TBI video addressed critical evaluation and ethical issues; these elements were also represented in the scoring rubric. The performance differences may therefore reflect both broader AI literacy development and immediate content-specific learning.

Fifth, the task-type manipulation was implemented through different levels of constraint in the generation goal. Although platform settings, procedures, and submission requirements were held constant, the manipulation cannot isolate every psychological consequence of task openness, such as perceived ambiguity or creative freedom. Future studies could experimentally separate these components and examine whether their effects depend on learner expertise or assessment criteria.

Sixth, relatedness was measured in an individually completed online activity with limited direct peer interaction. Future research should test collaborative tasks in which social connection can operate more directly.

Finally, the fsQCA configurations depend on the selected calibration anchors, frequency threshold, and consistency cutoff. Replication with independent samples and alternative calibration specifications is needed to assess configurational robustness.

## 6 Conclusion

This study demonstrates that instructional approach and task design make distinct and conditional contributions to university students’ AI literacy performance. Thinking-based instruction produced higher performance than skills-based instruction under both structured and open-ended tasks, with a larger advantage in the open-ended condition. Open-ended tasks also improved overall performance, particularly when accompanied by thinking-based instruction.

The psychological mechanisms were differentiated. Thinking-based instruction influenced performance indirectly through autonomy and competence, whereas task openness operated primarily through autonomy. Competence emerged as the strongest independent psychological predictor of performance, followed by autonomy. Relatedness was responsive to instructional design but did not independently predict immediate individual performance.

The configurational results further showed that high AI literacy can arise through multiple combinations of instructional, task, and psychological conditions. Effective AI literacy education therefore requires more than a single preferred teaching method. It requires alignment between instructional content, task demands, and the psychological resources needed for learners to exercise informed judgment, maintain competence, and engage autonomously with AI.

## Data Availability

The raw data supporting the conclusions of this article will be made available by the authors, without undue reservation.
