# Supplementary material for: Designing for AI literacy: psychological mechanisms and self-determination
Source: Front Psychol. 2026 Sep 14;17:1937845. doi: 10.3389/fpsyg.2026.1937845 (PMC13617005; doi:10.3389/fpsyg.2026.1937845)

Supplementary Material

This single Supplementary Material file consolidates Supplementary Tables S1–S3, the complete SBI and TBI instructional materials, the task-type manipulation materials, standardized submission procedures, Dreamina platform settings, and detailed experimental implementation referenced in the manuscript.

1 Supplementary Tables

Supplementary Table S1. Instructional-Video Alignment and Delivery Controls

Specification	SBI	TBI
Duration	6 min 28 s	6 min 30 s
Original intervention language	Mandarin Chinese	Mandarin Chinese
Narrator	Same Mandarin-speaking narrator	Same Mandarin-speaking narrator
Subtitles	Simplified Chinese	Simplified Chinese
Resolution	1920 × 1080	1920 × 1080
Audio	Voice-over only; no background music or sound effects	Voice-over only; no background music or sound effects
Playback controls	Fixed at 1.0×; fast-forwarding and speed adjustment disabled; pausing disabled during initial presentation; replay disabled after completion	Same controlled setting
Presentation / viewing	Full-screen; 100% minimum viewing requirement	Full-screen; 100% minimum viewing requirement
Completion recording	Start time, viewing progress, completion status, and page-exposure duration recorded	Same controlled setting
Instructional emphasis	Prompt structure, controllable visual settings, output inspection, quality control, and iterative revision	Generation mechanisms, model limitations, critical evaluation and verification, bias, copyright/privacy, and human judgment

Note. Full translated narration, chapter-level timelines, and chapter-specific instructional screens are provided in Supplementary Sections S1 (SBI) and S2 (TBI) below.

Supplementary Table S2. Measurement Properties of the Secondary AI-Literacy Self-Awareness Measures

Indicators	Loadings	Cronbach's Alpha	rho_A	Composite Reliability	AVE
**Conceptual Understanding**					
I have a general understanding of how the AIGC tools I use generate new images by learning from massive datasets of pictures.	0.884	0.851	0.857	0.914	0.776
I understand that "prompts" are key instructions that guide the AI to generate content.	0.898				
I am aware that the capabilities of AIGC tools are limited and that they are not omnipotent.	0.877				
**Critical Evaluation**					
I carefully examine the images generated by AIGC to see if they accurately reflect my requirements.	0.895	0.872	0.873	0.925	0.799
I contemplate potential errors or biases in the images generated by AIGC.	0.906				
I can assess the strengths and weaknesses of AIGC tools when generating images on specific themes.	0.883				

Note. Conceptual understanding and critical evaluation were retained as complementary self-awareness measures and were analyzed descriptively and correlationally rather than as mediators or fsQCA conditions. Their square roots of AVE were 0.881 and 0.894, respectively, and their intercorrelation was 0.504.

2 Supplementary Instructional Materials

2.1 Supplementary Section S1: Skills-Based Instruction (SBI)

SBI: Skills-Based Instruction

Operational definition. In this study, Skills-Based Instruction (SBI) was operationalized as instruction focused on concrete, observable procedures. Learners were shown a step-by-step workflow for performing the task, comparing generated outputs with an intended target, identifying specific mismatches, and revising controllable elements through repeated generation and self-evaluation. For AIGC image prompting, the SBI video focused on prompt structure, controllable visual settings, output inspection, quality control, and iterative revision.

Mastering AIGC Prompting: A Guide to Core Techniques is a 6 min 28 sec skills-oriented lesson centered on prompt structure, controllable visual settings, output inspection, quality control, and iterative revision.

Production and delivery controls for the SBI and TBI videos are summarized comparatively in Supplementary Table S1.

Seven-Chapter Skill Progression

1. Learning Objectives: Recognise the lesson goal: create an AIGC image and move from a basic description to deliberate visual direction.
2. Basic Prompt Structure: Use a reusable prompt scaffold: subject and details, style or mood, camera direction, action, and generation controls.
3. Subject and Detail Description: Expand a subject by specifying viewpoint, setting, light, colour, mood, action, and composition.
4. Style and Generation Controls: Control aesthetic and technical output by selecting style and genre terms and adjusting style, aspect ratio, image dimensions, composition, lighting, and other generation controls available in the experimental interface. Compare controlled variations to identify how each setting affects the result.
5. Exclusionary Guidance and Quality Control: Describe unwanted content or apply equivalent quality-control instructions for low quality, blur, anatomy errors, duplicate objects, and other defects, then inspect the result.
6. Iterative Optimisation: Generate, diagnose, revise one controllable element, compare variations, and repeat until the output meets the goal.
7. Integrated Example and Checklist: Transform a simple idea into a complete prompt, review all prompt components, generate, and decide what to revise next.

SBI Video and Narration Notes

Chapter 1 | Learning Objectives 0:00–0:30

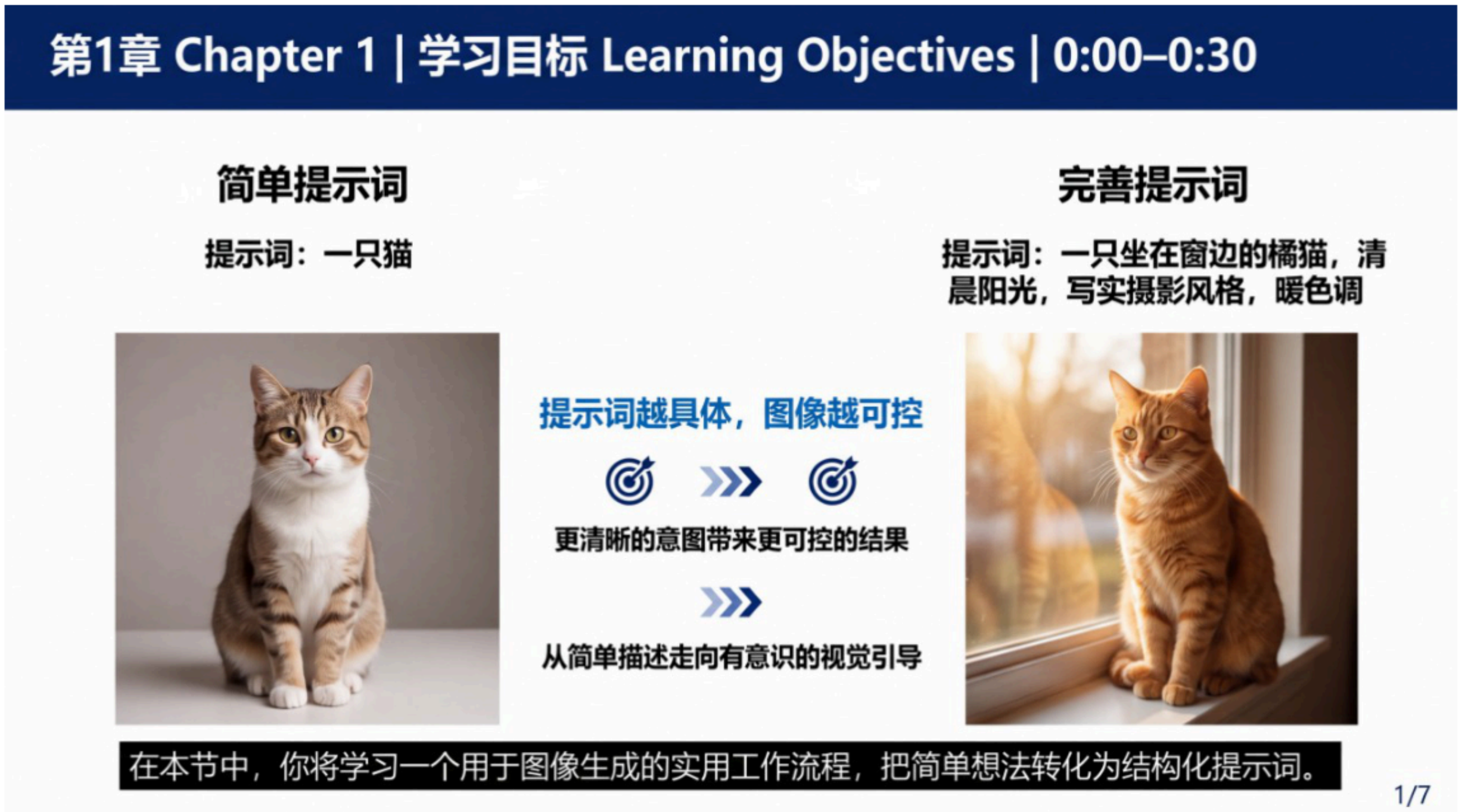

Supplementary Figure S1.1. SBI Chapter 1 instructional screen: Learning Objectives.

Narration (English translation of the original Mandarin intervention): Welcome to Mastering AIGC Prompting: A Guide to Core Techniques. In this lesson, you will learn a practical workflow for directing AI image generation. Compare the two examples on screen. A simple prompt such as "a cat" gives the system very little visual direction. By adding the setting, lighting, style, and colour tone, the intended image becomes much clearer. The goal is to turn a simple idea into a structured prompt that produces a more controllable result.

Chapter 2 | Basic Prompt Structure 0:30–1:20

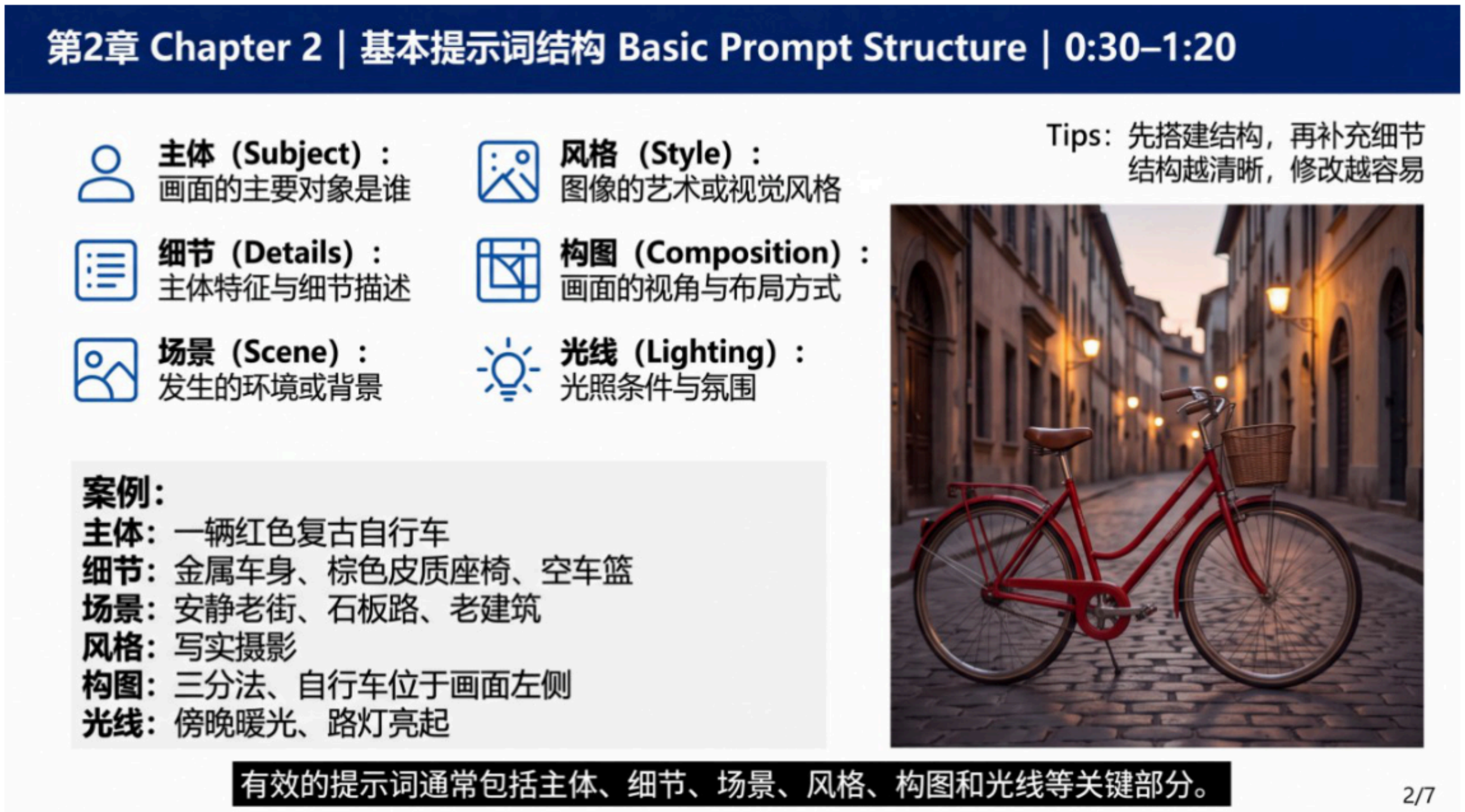

Supplementary Figure S1.2. SBI Chapter 2 instructional screen: Basic Prompt Structure.

Narration (English translation of the original Mandarin intervention): An effective image prompt usually contains several clear components. Start with the main subject, then add relevant details about its appearance or features. Next, describe the setting, visual style, composition, and lighting. For example, instead of simply asking for a red bicycle, we can specify a red vintage bicycle with a metal frame and brown leather seat, placed on a quiet old street at dusk. By organising the prompt into clear components, you can identify which part needs to be adjusted without rewriting the entire instruction.

Chapter 3 | Subject and Detail Description 1:20–2:30

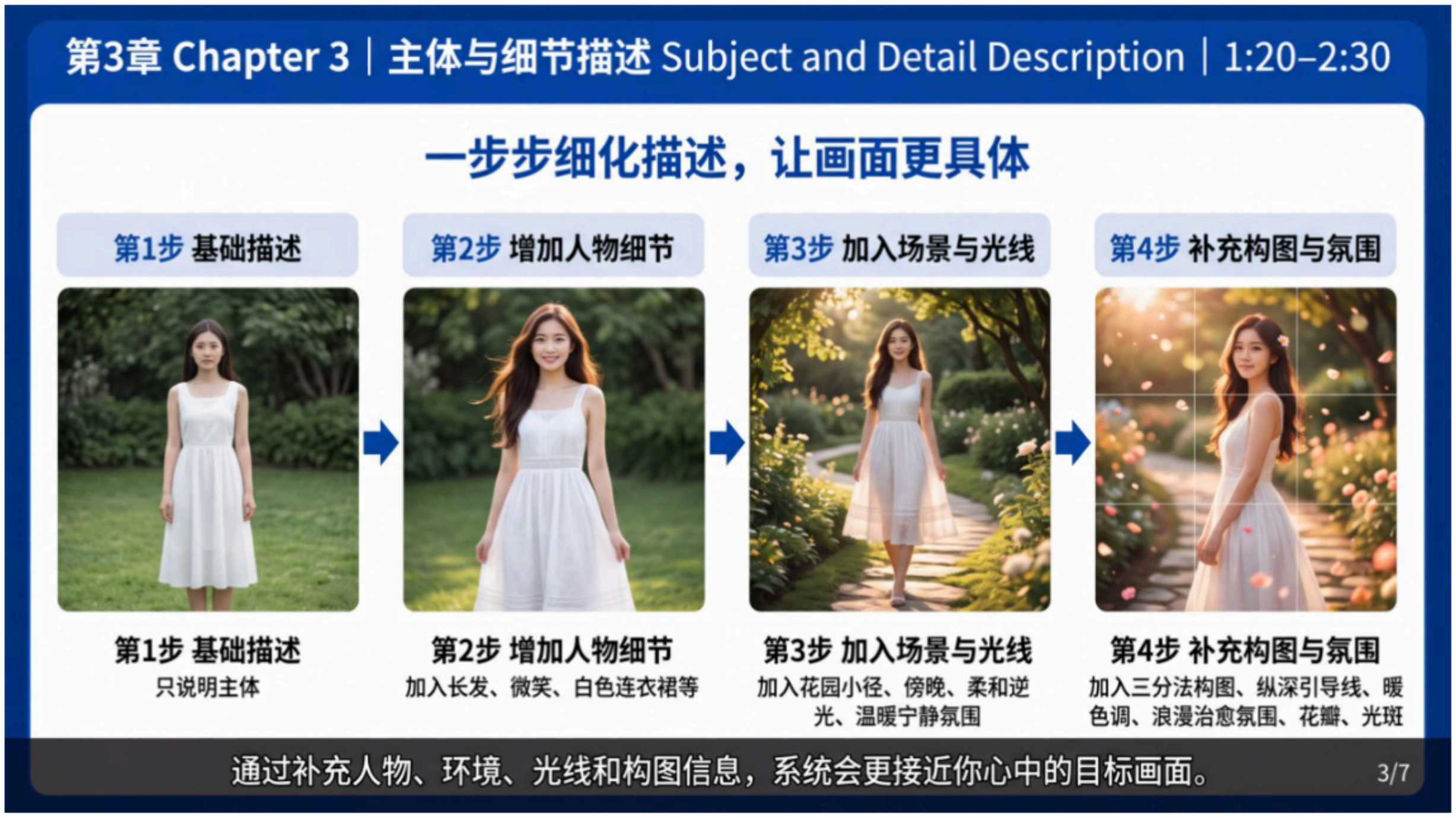

Supplementary Figure S1.3. SBI Chapter 3 instructional screen: Subject and Detail Description.

Narration (English translation of the original Mandarin intervention): Now let us refine the subject step by step. Begin with a basic description, such as a young woman standing in front of the camera. Then add useful details, including her hairstyle, clothing, expression, and pose. Next, place the subject in a specific environment and describe the time of day and lighting. In this example, the woman is placed on a garden path at sunset with soft backlighting. Finally, add composition, colour tone, and atmosphere. Each additional detail helps the system construct a scene that more closely matches your intended image.

Chapter 4 | Style and Generation Controls 2:30–3:25

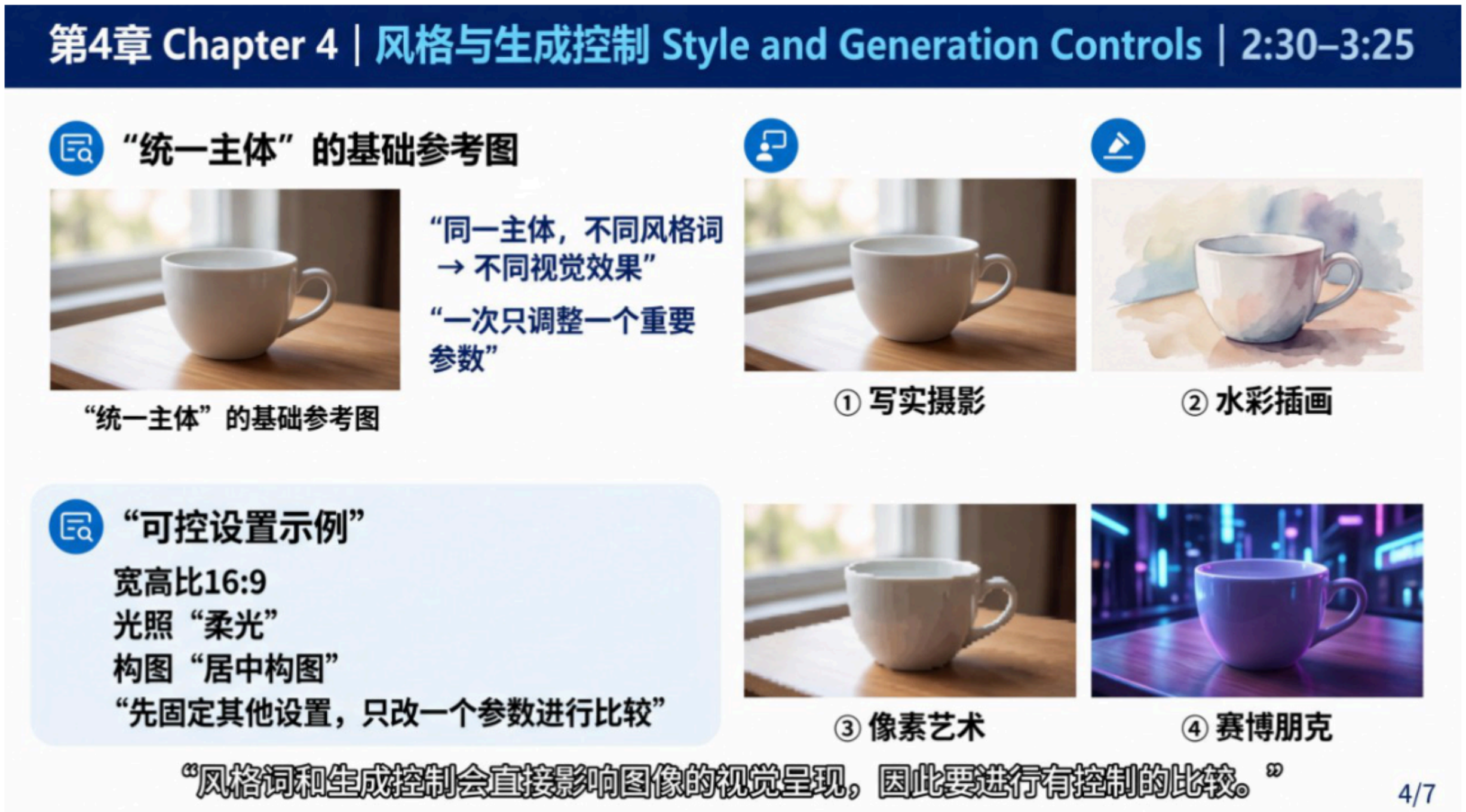

Supplementary Figure S1.4. SBI Chapter 4 instructional screen: Style and Generation Controls.

Narration (English translation of the original Mandarin intervention): Once the subject and content are clear, you can control how the image looks. Here, the same ceramic cup is generated in four different visual styles: realistic photography, watercolor illustration, pixel art, and cyberpunk. The subject remains the same, but changing the style term produces very different visual results. Other controllable settings, such as aspect ratio, lighting, and composition, can also affect the output. When comparing results, keep the other settings stable and change only one important element at a time. This makes the effect of each adjustment easier to identify.

Chapter 5 | Exclusionary Guidance and Quality Control 3:25–4:15

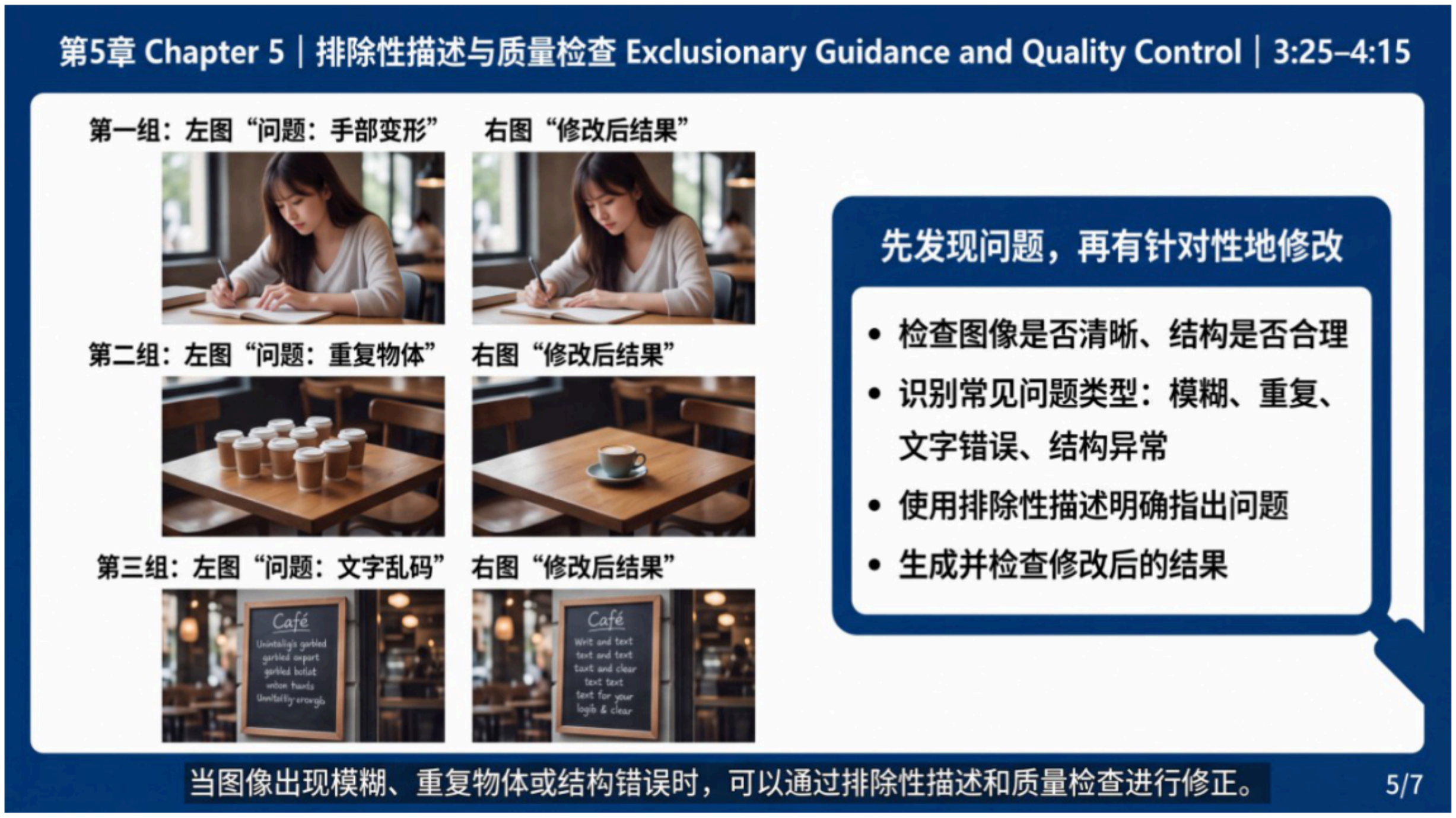

Supplementary Figure S1.5. SBI Chapter 5 instructional screen: Exclusionary Guidance and Quality Control.

Narration (English translation of the original Mandarin intervention): After generating an image, inspect the result for visible problems. Common defects may include distorted hands, duplicated objects, blurred details, incorrect text, or other structural errors. In the examples shown here, the first images contain specific defects, while the revised images show improved results. Identify the problem clearly before making a change. Where the interface supports it, use exclusionary instructions such as avoiding duplicated objects or distorted anatomy. Otherwise, use the same criteria as a quality-control checklist, revise the prompt, generate again, and inspect the new result.

Chapter 6 | Iterative Optimisation 4:15–5:40

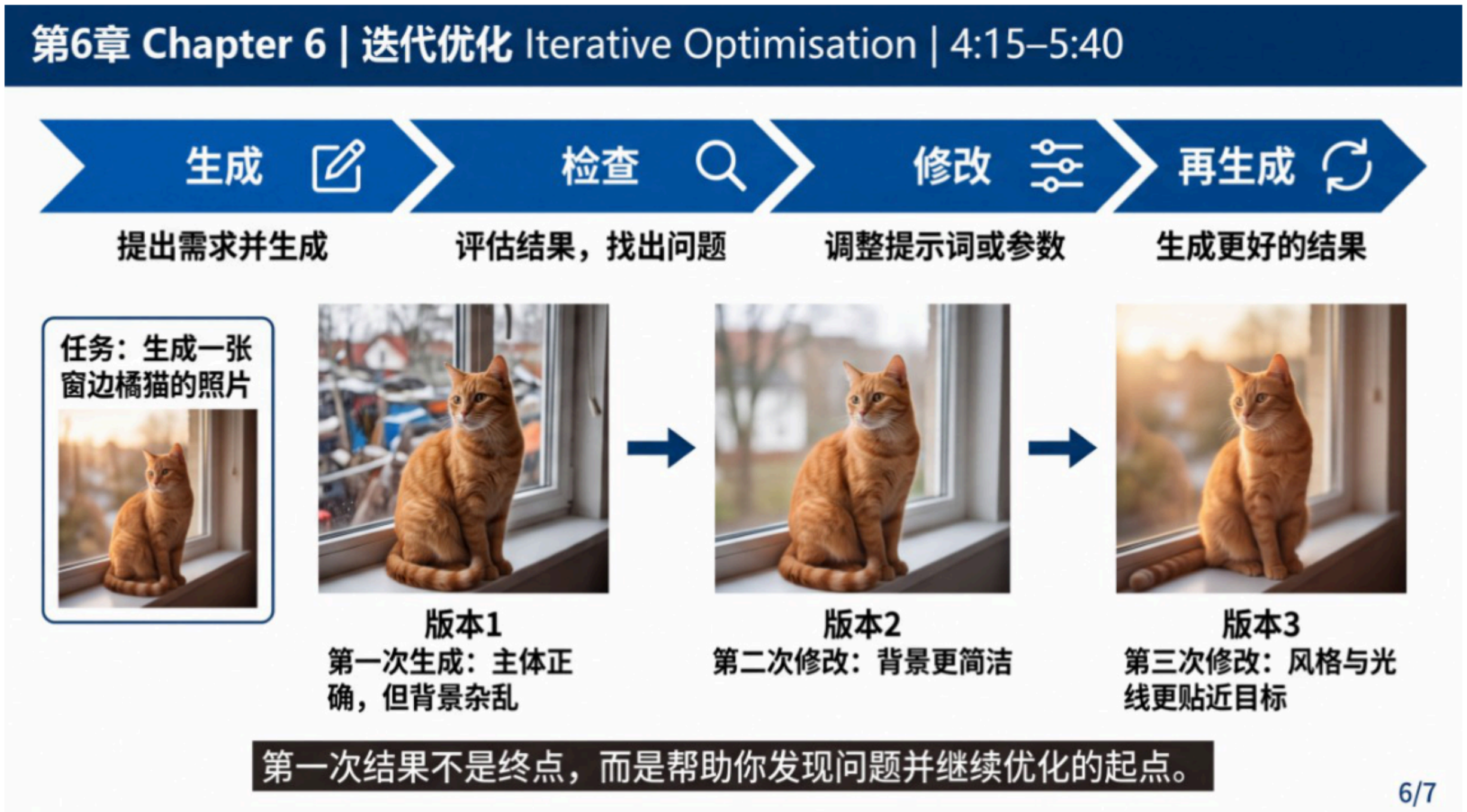

Supplementary Figure S1.6. SBI Chapter 6 instructional screen: Iterative Optimisation.

Narration (English translation of the original Mandarin intervention): Good prompting usually requires more than one generation. Treat the first result as a starting point for revision. In this example, the task is to create a photograph of an orange cat sitting beside a window. The first version contains the correct subject, but the background is too busy. In the second version, the background is simplified. In the third version, the lighting and visual style are adjusted to better match the target image. The workflow is simple: generate, inspect the result, identify one clear problem, revise one controllable element, and generate again. Comparing versions helps you understand which change improved the image.

Chapter 7 | Integrated Example and Checklist 5:40–6:28

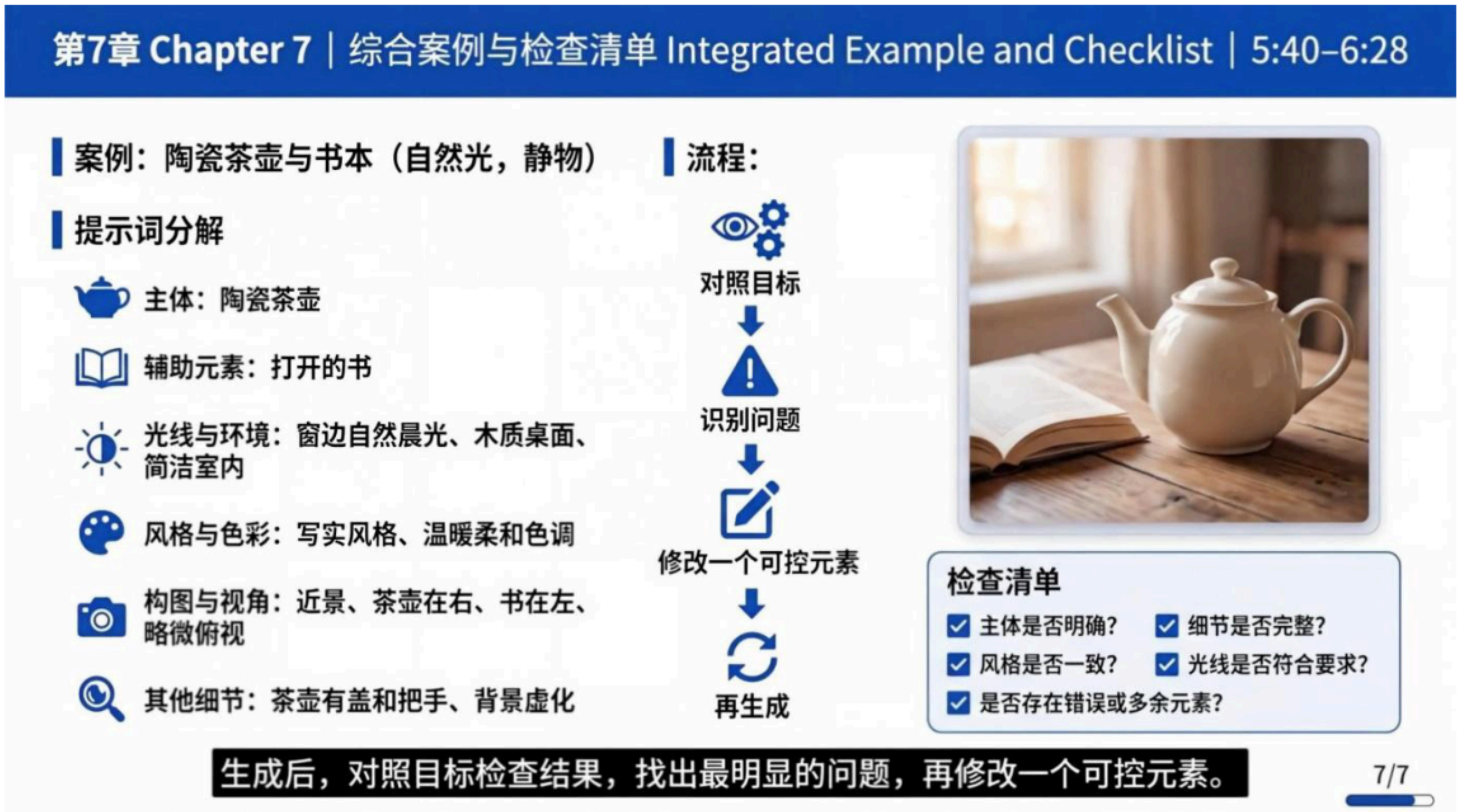

Supplementary Figure S1.7. SBI Chapter 7 instructional screen: Integrated Example and Checklist.

Narration (English translation of the original Mandarin intervention): Let us bring the workflow together with a complete example. The main subject is a ceramic teapot beside an open book on a wooden table. We add soft morning light from a nearby window, a realistic photographic style, a warm colour tone, and a close composition with the teapot on the right and the book on the left. After generating the image, compare the result with the intended visual requirements. Check whether the subject is clear, the details are complete, the style and lighting are consistent, and whether any unwanted elements remain. Identify the most important mismatch, revise one controllable element, and generate again if necessary.

2.2 Supplementary Section S2: Thinking-Based Instruction (TBI)

TBI: Thinking-Based Instruction

Operational definition. In this study, Thinking-Based Instruction (TBI) was operationalized as instruction focused on explicit reasoning, evaluation, and reflective judgment. Learners were guided to understand how AIGC image-generation systems work, identify their limitations, evaluate generated outputs, recognise potential bias and risks, and make informed judgments about their appropriate use. For AIGC image generation, the TBI video focused on generation mechanisms, model limitations, critical evaluation and verification, bias and stereotypes, copyright and privacy, and human judgment in the final use of generated images.

A Collaborative Dance with AI: Understanding and Critically Using Image Generation Tools is a 6 min 30 sec thinking-oriented lesson centered on generation mechanisms, model limitations, critical evaluation and verification, bias and stereotypes, copyright and privacy, and human judgment in the final use of generated images.

Production and delivery controls for the SBI and TBI videos are summarized comparatively in Supplementary Table S1.

Seven-Chapter Thinking Progression

1. Learning Objectives: Understand that effective use of AIGC image tools involves explanation, evaluation, verification, and human judgment rather than tool operation alone.
2. How AIGC Generates Images: Understand image generation as a probabilistic process in which visual patterns are gradually constructed from learned image–text relationships.
3. Limits of Image-Generation Systems: Recognise that visually convincing images may contain fabricated details, spatial inconsistencies, incorrect text, or other errors because the system does not understand reality in the same way humans do.
4. Critical Evaluation of Generated Images: Evaluate generated images by comparing them with trusted information and matching the level of verification to the intended use of the image.
5. Bias and Stereotypes: Identify repeated differences and stereotypical patterns in generated images and consider how such patterns may arise from training data and existing media representations.
6. Copyright, Privacy, and Responsibility: Consider copyright, personal-data risks, attribution, and the role of human decisions in the use and distribution of generated images.
7. Human–AI Collaboration and Reflection: Use AI generation as part of a human-led process in which people define goals, evaluate alternatives, check limitations, and make final judgments.

TBI Video and Narration Notes

Chapter 1 | Learning Objectives | 0:00–0:35

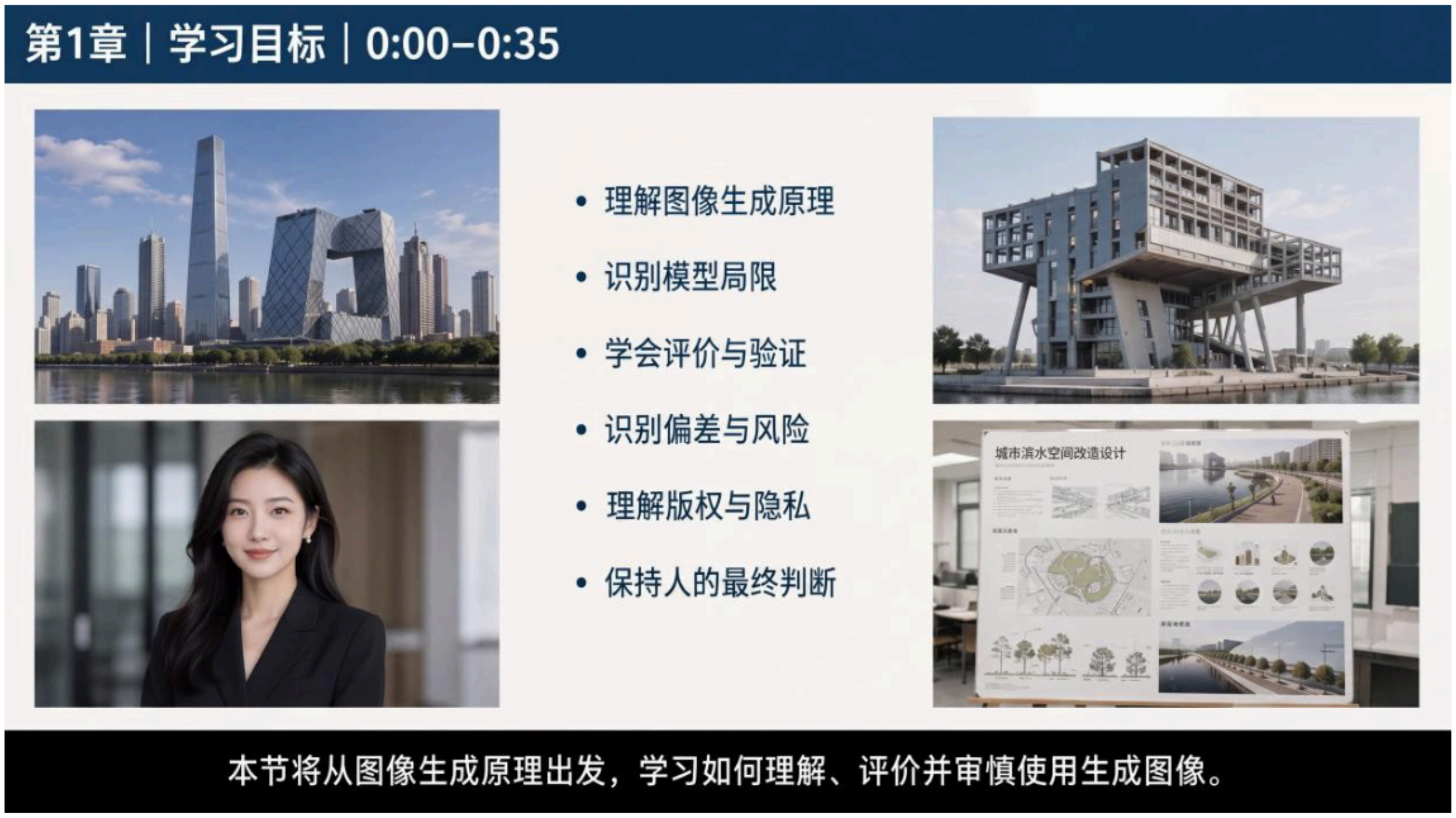

Supplementary Figure S2.1. TBI Chapter 1 instructional screen: Learning Objectives.

Narration (English translation of the original Mandarin intervention): Welcome to A Collaborative Dance with AI: Understanding and Critically Using Image Generation Tools. In this lesson, we will move beyond simply operating an image generator. We will examine how generated images are produced, where AI can fail, how bias and risk may enter the process, and why human judgment remains important. The goal is to understand, evaluate, verify, and reflect on generated images rather than accept them at face value. Let us begin with a basic question: how does an AI system generate an image?

Chapter 2 | How AIGC Generates Images | 0:35–1:35

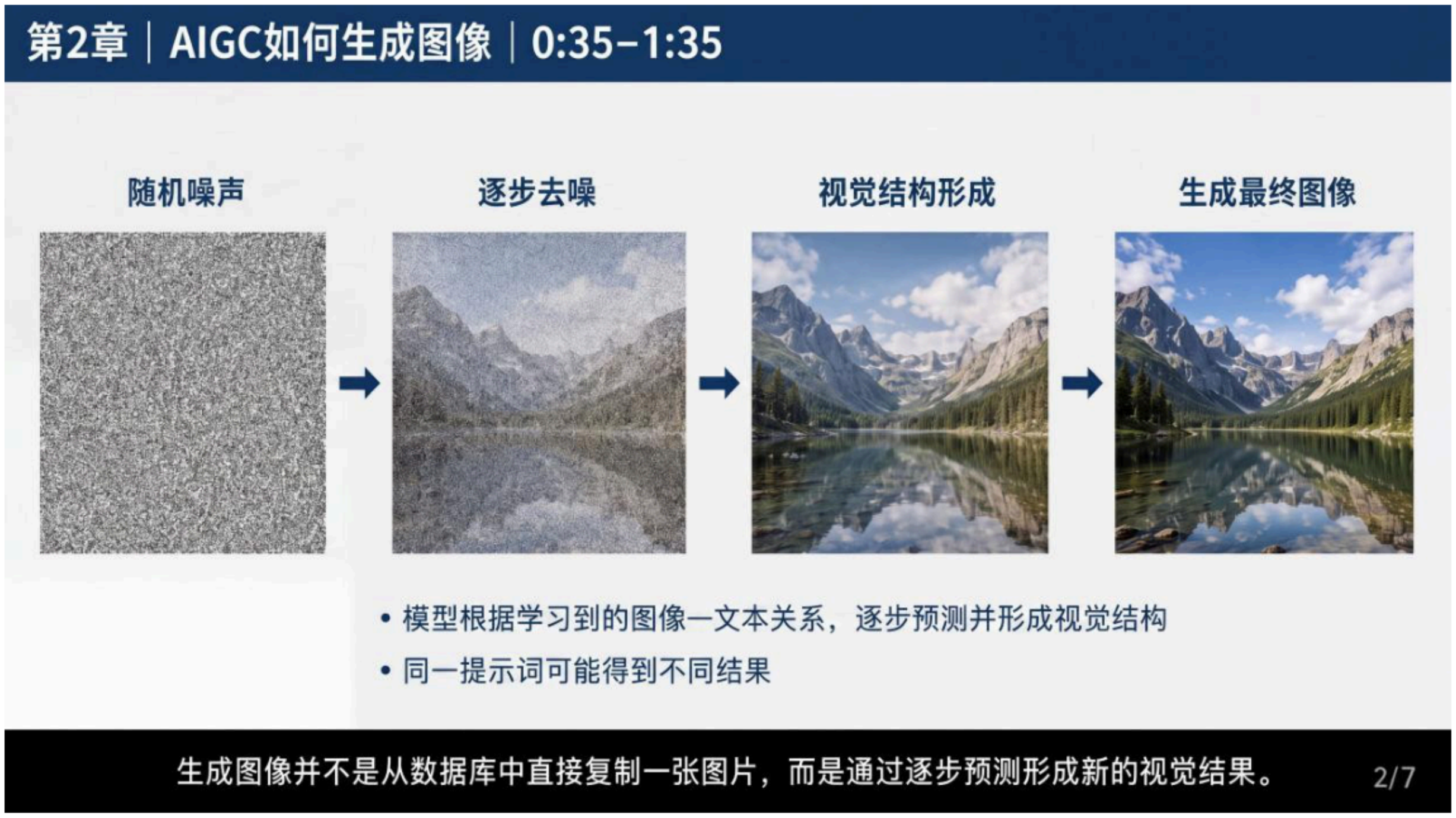

Supplementary Figure S2.2. TBI Chapter 2 instructional screen: How AIGC Generates Images.

Narration (English translation of the original Mandarin intervention): It does not draw in the same way as a human artist, nor does it understand an object through lived experience. Instead, it learns statistical patterns from large collections of data. In a diffusion-based process, an image can first be represented through increasing levels of noise, and the model learns how to reverse that process. Starting from random noise, it repeatedly predicts what should be removed or adjusted, gradually forming shapes, colors, textures, and relationships that correspond to the prompt. Each step is based on probability rather than certainty, which is why the same prompt can produce different results. The final image is therefore not retrieved as a complete picture from memory. It is constructed step by step through a learned process of probabilistic denoising.

Chapter 3 | Limits of Image-Generation Systems | 1:35–2:30

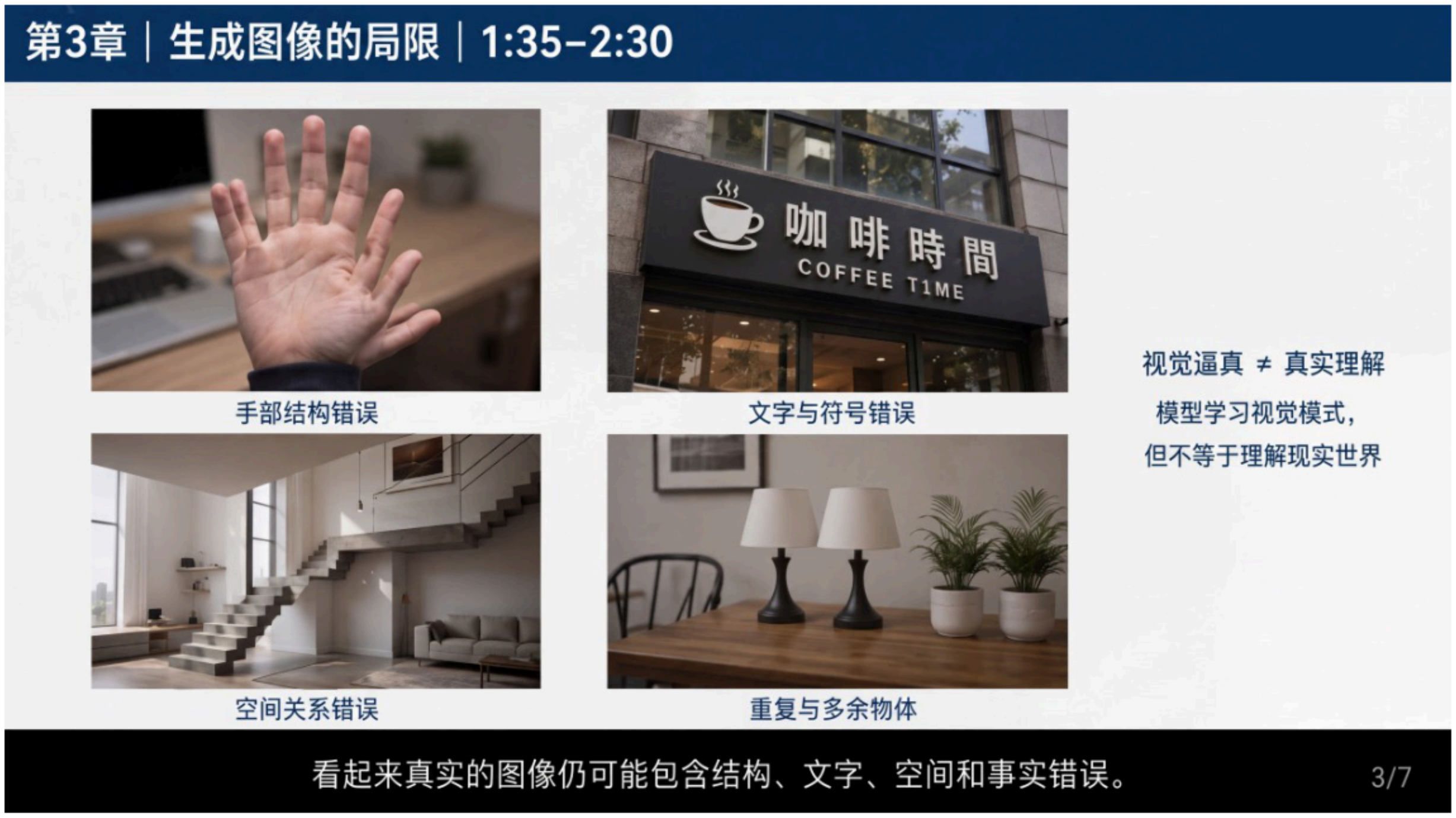

Supplementary Figure S2.3. TBI Chapter 3 instructional screen: Limits of Image-Generation Systems.

Narration (English translation of the original Mandarin intervention): This generation process also helps explain important limitations. A visually convincing image does not mean that the system understands reality. Image-generation systems predict likely visual patterns from learned data, but they may not understand objects, spatial relationships, text, or human intentions in the way people do. As a result, they can fabricate details, combine incompatible elements, distort anatomy, or produce plausible-looking but incorrect information. When examining a generated image, ask not only whether it looks realistic, but also what may be wrong, missing, or inconsistent.

Chapter 4 | Critical Evaluation of Generated Images | 2:30–3:30

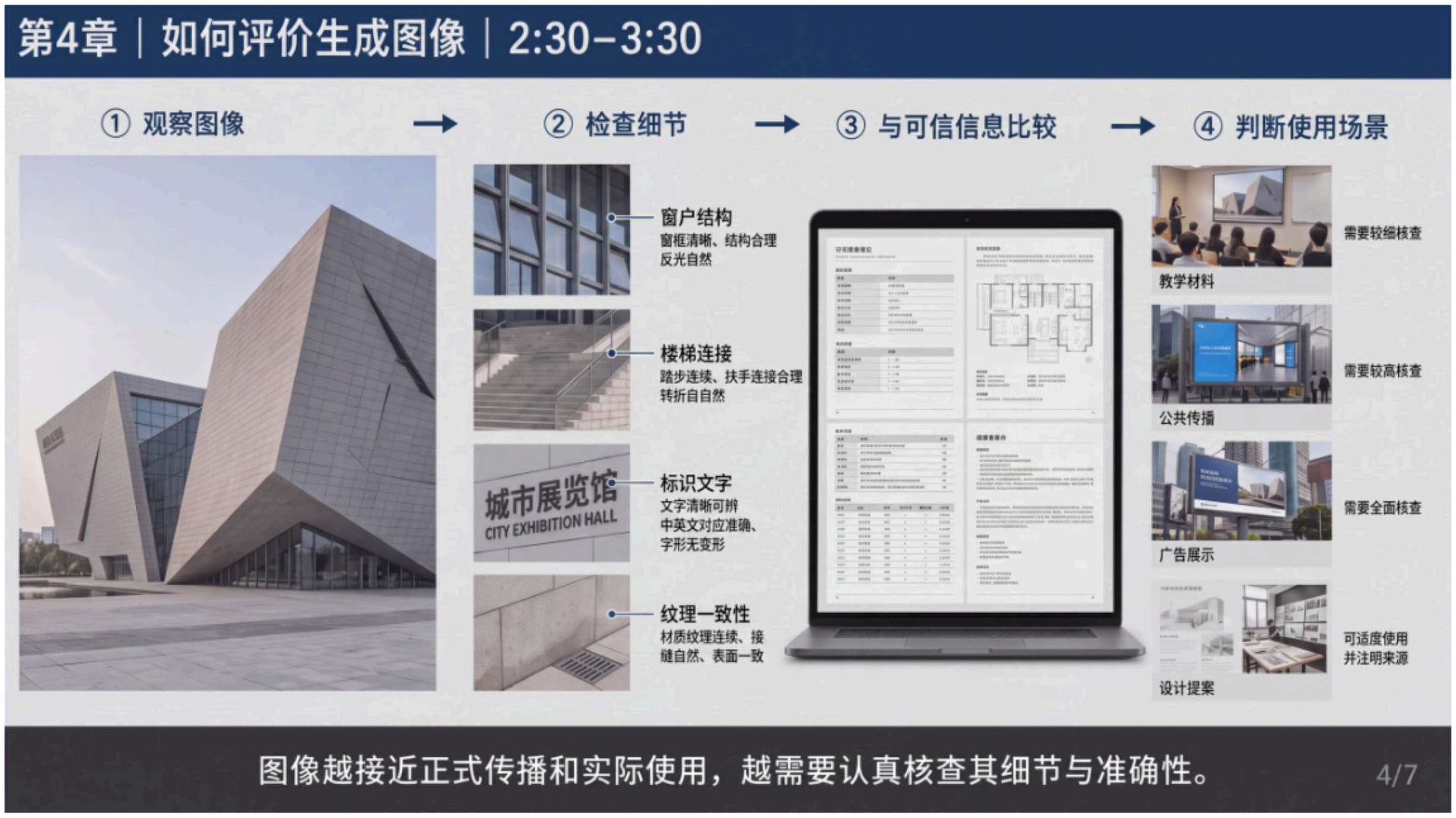

Supplementary Figure S2.4. TBI Chapter 4 instructional screen: Critical Evaluation of Generated Images.

Narration (English translation of the original Mandarin intervention): This question leads us to the next stage: critical evaluation. Polished imagery is not evidence of accuracy. We should compare generated results with trusted sources, inspect whether important details are consistent, and consider how much time and expertise are required to verify them. Technical improvements may reduce errors, but they do not replace professional judgment. A designer must still assess visual meaning, and a teacher must judge whether an image is educationally appropriate. The consequences of an error depend on how an image is used. A distorted detail in an early creative sketch may be easy to correct, whereas an inaccurate image used in public communication, educational materials, advertising, or a design presentation may mislead its audience. Verification should therefore be matched to the intended use and potential impact of the generated image.

Chapter 5 | Bias and Stereotypes | 3:30–4:25

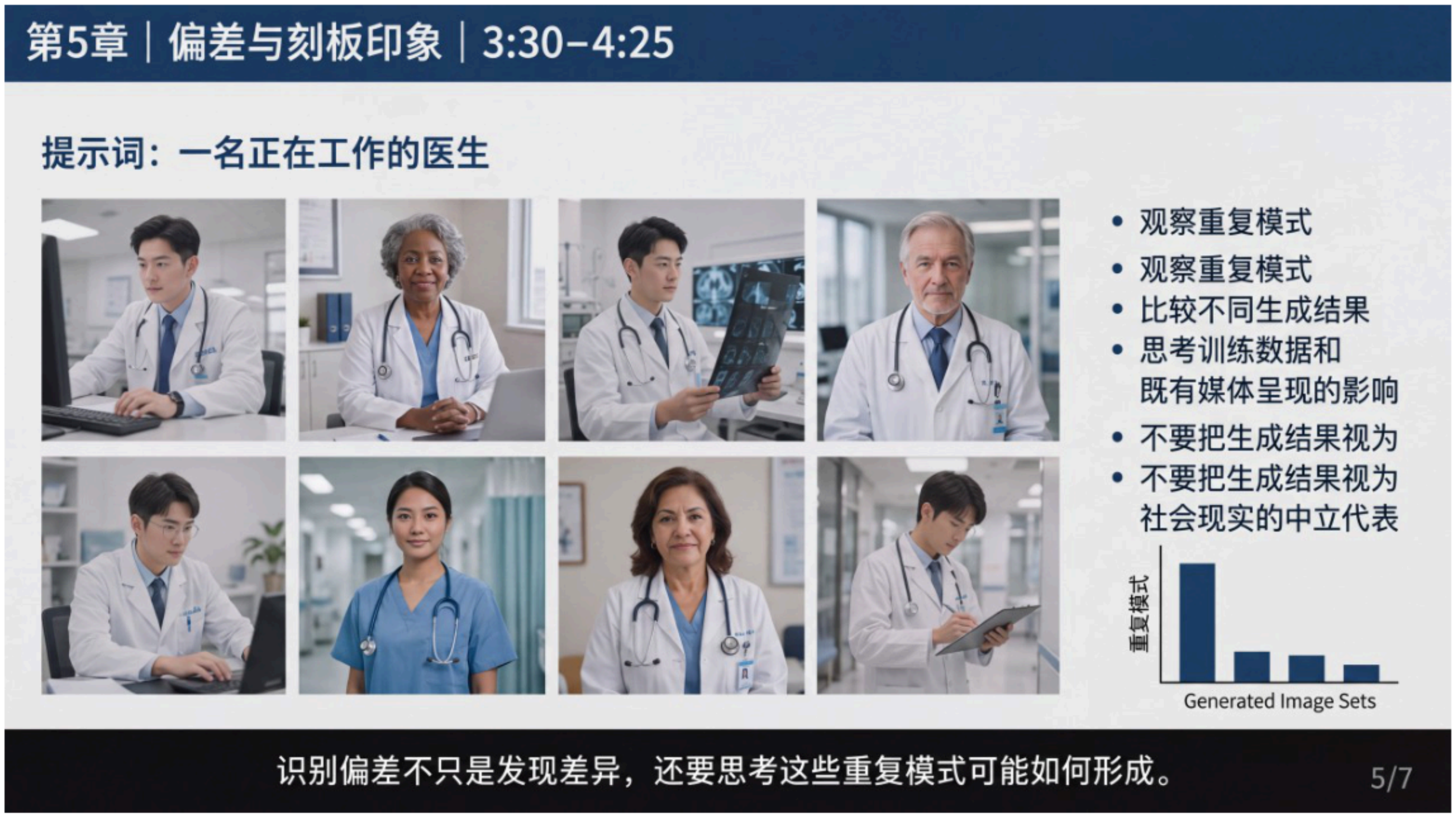

Supplementary Figure S2.5. TBI Chapter 5 instructional screen: Bias and Stereotypes.

Narration (English translation of the original Mandarin intervention): Another important issue is bias. Bias can be examined through several layers of evidence. First, we can compare outputs across different prompts, social groups, or platforms and look for repeated differences. Second, we can inspect the images themselves for stereotypical roles, appearances, settings, or behaviors. Third, we can ask where these patterns may come from. Training data reflect existing media, cultural assumptions, and unequal patterns of visibility. When these patterns are repeatedly learned and reproduced at scale, the system may preserve or even amplify them. For example, certain occupations may be associated mainly with one gender, age group, or cultural background. The task is therefore not only to notice that outputs are different. We should explain how these differences may have formed, test whether they are systematic, and avoid treating generated images as neutral representations of society.

Chapter 6 | Copyright, Privacy, and Responsibility | 4:25–5:15

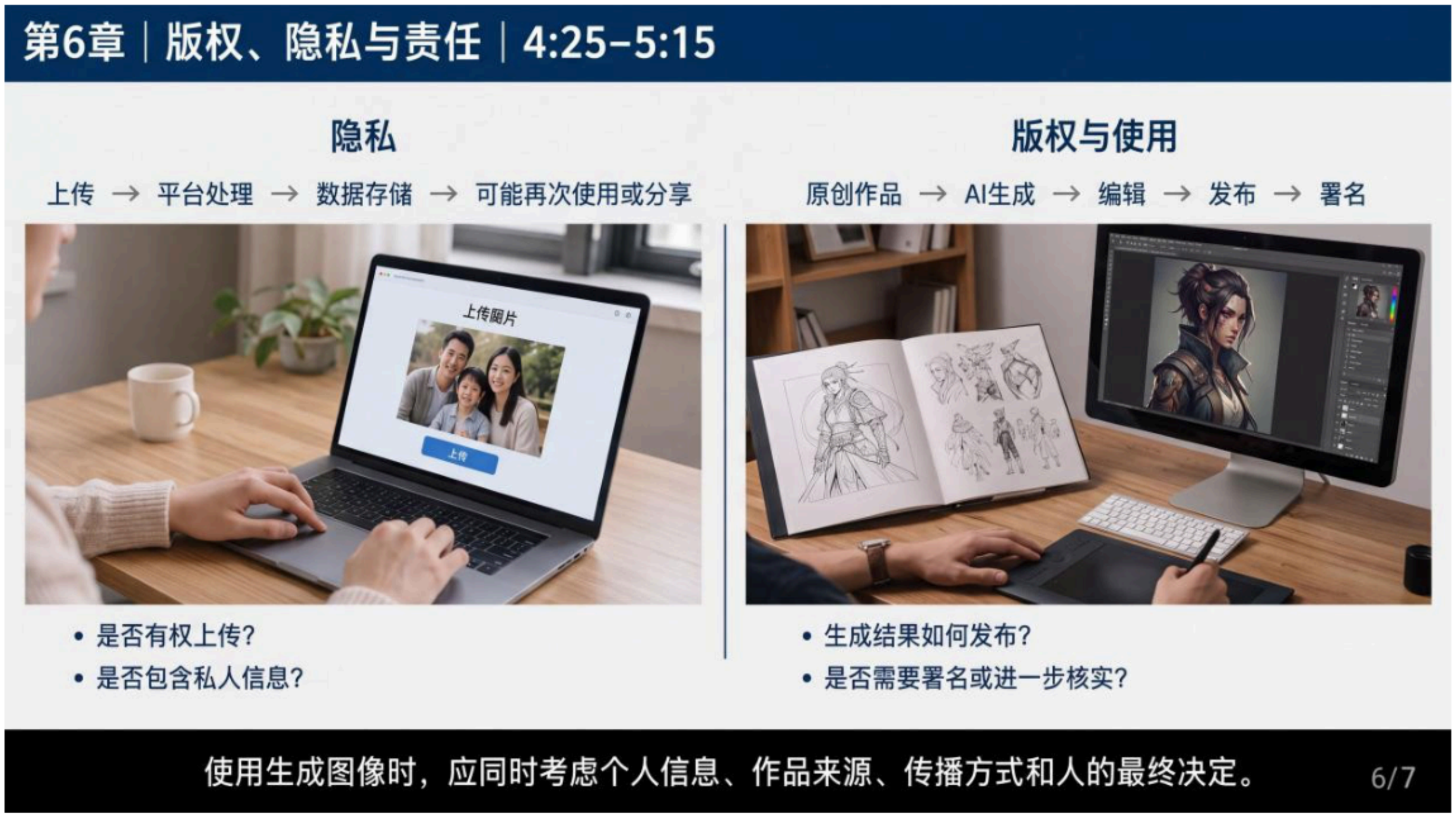

Supplementary Figure S2.6. TBI Chapter 6 instructional screen: Copyright, Privacy, and Responsibility.

Narration (English translation of the original Mandarin intervention): Responsible use also requires attention to copyright and privacy. Generated images may be influenced by protected works, and legal and ethical judgments depend on context. Users still make decisions about prompting, selection, editing, publication, and attribution. Privacy raises similar concerns. Personal photographs, private documents, or identifiable information should not be uploaded without careful consideration. Before using a platform, ask what information is being provided, how it may be stored or reused, and what risks may follow. The key point is that using an AI tool does not remove the need for careful human judgment about source material, personal data, and publication.

Chapter 7 | Human–AI Collaboration and Reflection | 5:15–6:30

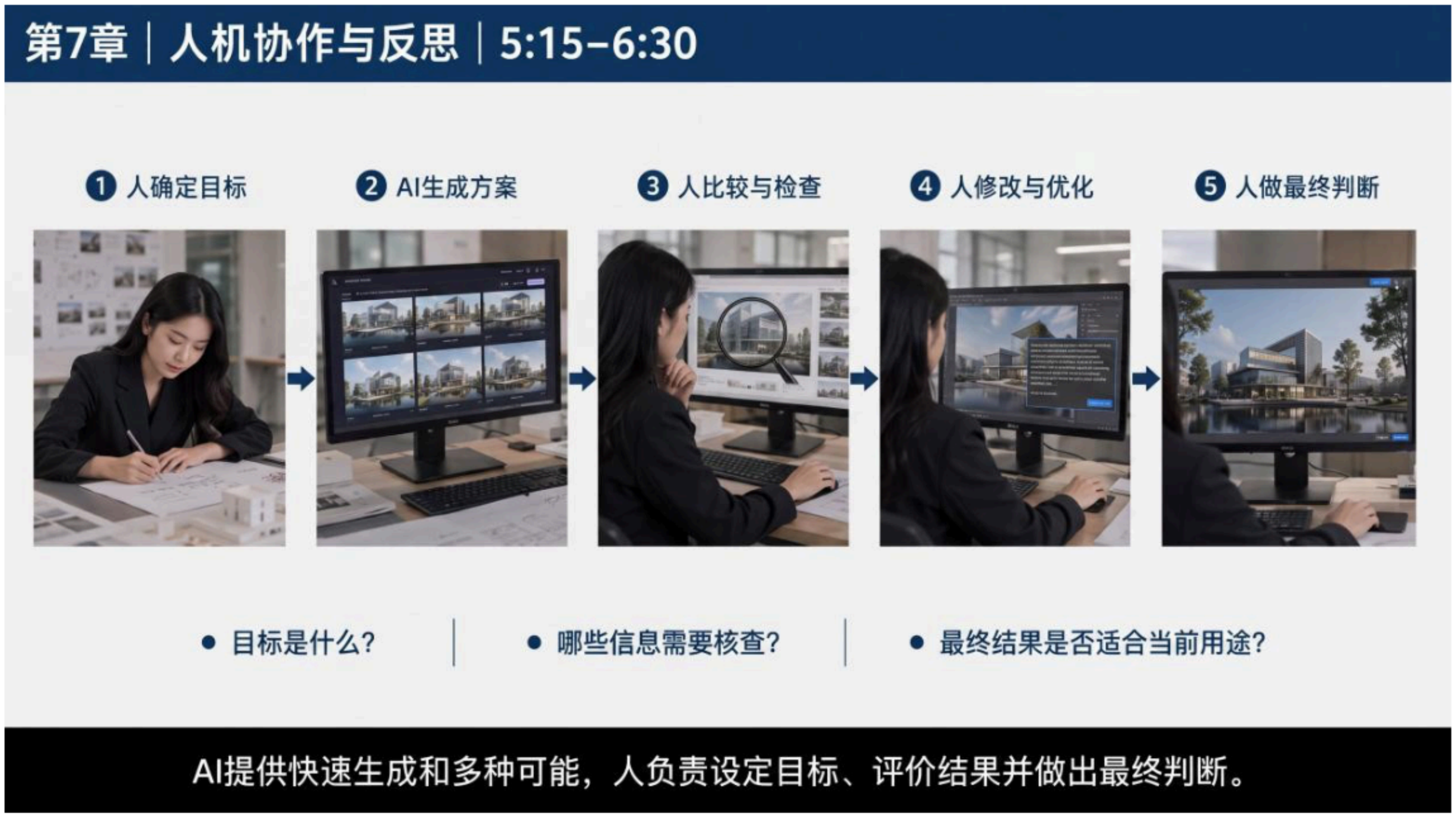

Supplementary Figure S2.7. TBI Chapter 7 instructional screen: Human–AI Collaboration and Reflection.

Narration (English translation of the original Mandarin intervention): What, then, does responsible human-AI collaboration look like? A useful workflow begins with people defining the goal. We decide what problem needs to be solved, what values matter, and what constraints must be respected. AI can then generate alternatives quickly and help us explore different compositions, styles, or creative directions, but generation is only one stage of the process. People must compare the alternatives, question their assumptions, check for errors and bias, and edit the results. We must also decide whether an output is appropriate for its intended audience and context. Most importantly, people must accept responsibility for the meaning, quality, and consequences of the final work. This relationship is better understood as collaboration rather than replacement. AI contributes speed, variation, and pattern generation. Humans contribute purpose, context, ethics, professional knowledge, and accountability. The quality of the final result depends on how carefully these different strengths are combined. As you use image generation tools, pause and ask yourself: What goal did I define? What evidence supports this output? What information might be missing or distorted? Who could be affected by the result? And am I prepared to take responsibility for using it? Critical and creative thinking begin when we stop asking only what AI can produce, and start asking how, why, and whether it should be used.

3 Supplementary Section S3: Task-Type Materials and Experimental Implementation

S3.1 Overview of the Task-Type Manipulation

All participants completed an AIGC image-generation task using Dreamina. Task type was experimentally manipulated by varying the degree of constraint imposed on the image-generation goal.

Participants assigned to the Structured Task (STT) condition were required to satisfy a predefined set of visual and stylistic criteria, whereas participants assigned to the Open-Ended Task (OET) condition were provided with a broad conceptual goal and were given greater freedom to determine the specific content, composition, and visual expression of the final image.

All other platform, procedural, and submission conditions were held constant across the two task conditions.

S3.2 Common Task-Introduction Screen

Original Mandarin instructions

AIGC 图像生成任务

接下来，你将使用 Dreamina 完成一项 AIGC 图像生成任务。

请仔细阅读下一页面所显示的具体任务要求，并根据任务要求独立完成图像创作。

在任务过程中，你可以根据生成结果修改和优化文字提示词、重新生成图像，并在需要时使用平台提供的局部重绘功能。请认真比较不同版本的生成结果，并从中选择一幅你认为最符合任务要求的图像作为最终作品。

完成任务后，你需要提交以下材料：

· 最终生成图像；
· 生成最终图像时使用的完整提示词；
· 图像生成过程说明，包括主要修改内容；
· 选择该图像作为最终作品的理由。

图像生成阶段限时 10 分钟。

在任务期间，请独立完成实验，不得使用其他生成式 AI 工具、搜索引擎或提示词网站，也不得与其他参与者讨论任务内容。

点击· 开始任务· 后进入正式任务阶段。

English translation

AIGC Image-Generation Task

You will now complete an AIGC image-generation task using Dreamina. Please carefully read the condition-specific instructions presented on the following page and complete the task independently.

During the task, you may revise and optimize your text prompts, regenerate images, and use the platform's inpainting/local-redrawing function when necessary. You should compare the different outputs and select the image that you believe best satisfies the assigned task as your final submission.

After completing the task, you will be required to submit the final generated image, the complete prompt used to generate the selected final image, a brief description of the image-generation process and major revisions, and the rationale for selecting the final image.

The image-generation stage lasts 10 minutes. During the task, you must work independently. The use of other generative-AI tools, search engines, or prompt-sharing websites is not permitted, and communication with other participants regarding the task is prohibited.

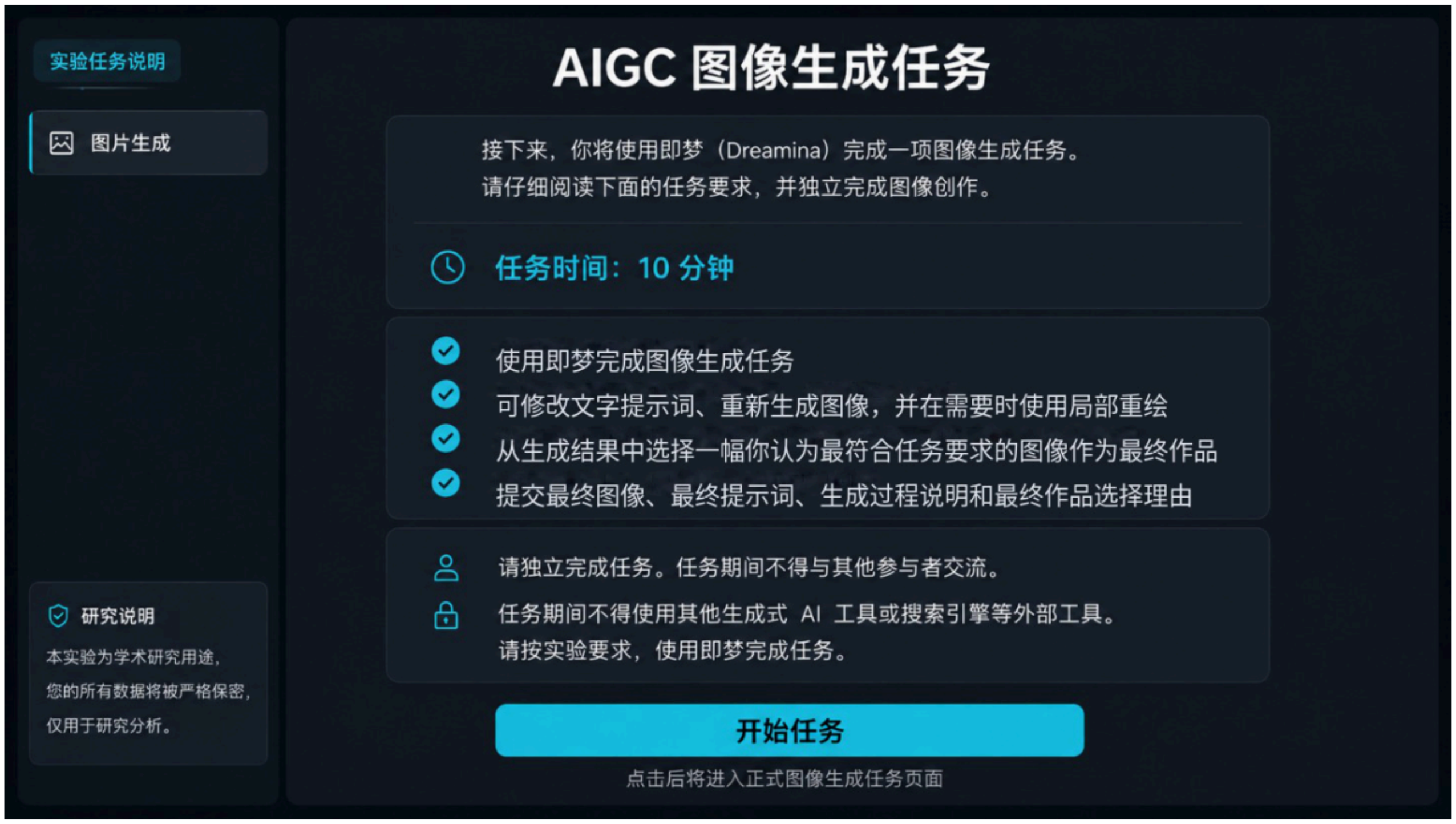

Supplementary Figure S3.1. Common task-introduction screen presented to all participants.

S3.3 Structured Task (STT)

Original Mandarin instructions

结构化任务：未来可持续城市

请使用 Dreamina 生成一幅主题为· 未来可持续城市· 的图像。

最终生成的图像必须同时包含以下内容：

1. 配有太阳能板的建筑；
2. 风力发电设施；
3. 清澈的河流；
4. 晴朗、明亮的环境；
5. 写实摄影风格。

在生成过程中，你可以根据当前生成结果调整文字提示词，并重新生成图像。

请比较不同版本的结果，并选择一幅最符合以上任务要求的图像作为最终作品。

完成后，请提交最终生成图像、最终提示词、图像生成过程说明及最终作品选择理由。

English translation

Structured Task: Sustainable City of the Future

Participants were instructed to use Dreamina to generate an image depicting a “Sustainable City of the Future.” The final image was required to include buildings equipped with solar panels, wind-power facilities, a clear river, a sunny and bright atmosphere, and a photorealistic visual style.

Participants could revise their text prompts in response to the generated outputs and generate additional versions during the task. They were instructed to compare the generated alternatives and select the image that best satisfied the predefined criteria as their final output.

After completing the task, participants submitted the final generated image, the final prompt, a description of the image-generation process, and the rationale for selecting the final image.

S3.4 Open-Ended Task (OET)

Original Mandarin instructions

开放式任务：人与自然和谐共生的未来

请使用 Dreamina 创作一幅能够表达· 人与自然和谐共生的未来· 这一概念的图像。

本任务不规定必须出现的具体人物、建筑、自然元素、构图或视觉风格。

你可以充分发挥想象，自主决定画面内容、构图、色彩、视觉风格以及具体表达方式。

请通过图像表达你对这一主题的理解。

完成后，请选择一幅最能够表达你的设计理念的图像作为最终作品，并提交最终生成图像、最终提示词、图像生成过程说明及最终作品选择理由。

English translation

Open-Ended Task: A Future of Harmonious Coexistence between Humans and Nature

Participants were instructed to use Dreamina to create an image expressing the concept of “a future of harmonious coexistence between humans and nature.”

No specific objects, scenes, compositions, or visual styles were prescribed. Participants were encouraged to use their imagination and independently determine the content, composition, colour scheme, and visual expression of the image.

Participants could revise their prompts and regenerate images during the task and selected the output that best represented their intended concept as their final submission.

After completing the task, participants submitted the final generated image, the final prompt, a brief description of the image-generation process, and the rationale for selecting the final output.

S3.5 Standardized Submission Form

To ensure comparability of the evidence available for AI literacy performance scoring, participants in both task conditions submitted the same core materials. The task-type manipulation therefore concerned the degree of constraint imposed on the generation goal rather than the quantity or type of evidence available for performance assessment.

1. Final Generated Image

Participants uploaded the single image selected as their final task output.

2. Final Prompt

Participants entered the complete text prompt used to generate the selected final image.

3. Image-Generation Process Statement

Participants briefly described the major revisions made during image generation, including what was changed, why it was changed, and how the generated result changed after revision.

4. Final-Output Selection Rationale

Participants briefly explained why they selected the submitted image as their final output and how the image satisfied the assigned task goal.

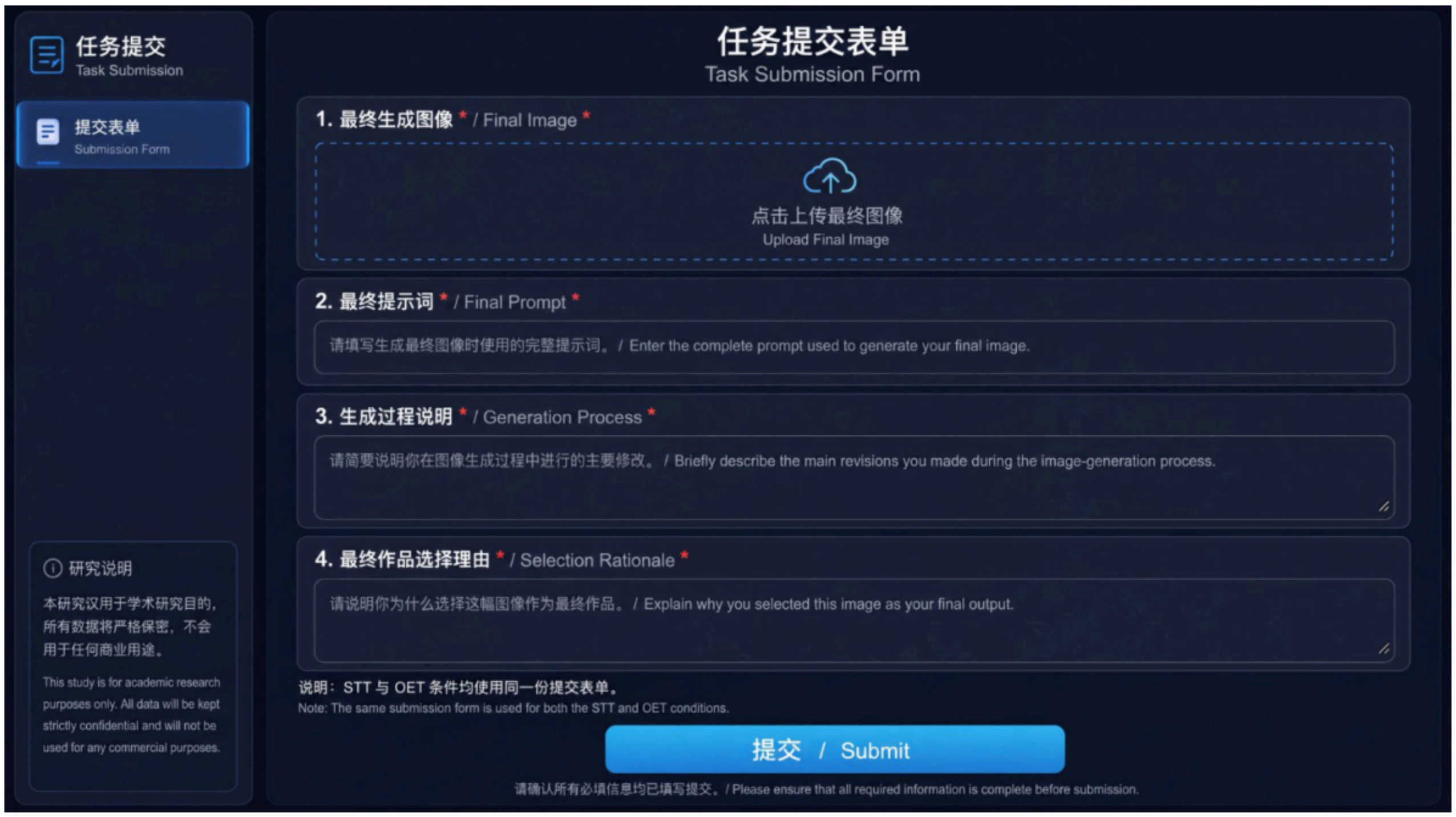

Supplementary Figure S3.2. Standardized task-submission form used in both task conditions.

S3.6 Dreamina Platform Settings and Experimental Restrictions

Supplementary Table S3. Dreamina Platform Settings and Experimental Restrictions

Specification	Experimental Setting
Platform	Dreamina
Access mode	Web-based version
Experimental data-collection period	15 February 2026–20 April 2026
Displayed model/version at the time of data collection	Image 5.0 lite
Account configuration	Same standardized account configuration for all participants
Generation mode	Text-to-image generation
Aspect ratio	3:4
Output resolution	768 × 1024 px
Candidate images per generation request	4
Maximum generation requests per participant	5 requests (4 candidate images per request; up to 20 candidate images in total)
Image-generation stage time limit	10 min
Reference-image upload	Not allowed
Automatic prompt enhancement	Not allowed
In-platform inpainting/local redrawing	Allowed

Specification	Experimental Setting
Other post-generation image editing	Not allowed
External search engines	Not allowed
Prompt-sharing websites	Not allowed
Other generative-AI tools	Not allowed
Communication with other participants	Not allowed
Completion mode	Individual
Required submission	Final image + final prompt + process statement + final-output selection rationale

Note. All platform settings and restrictions listed in Supplementary Table S3 were applied identically across the four experimental conditions. The experimental manipulations concerned only instructional approach (SBI vs. TBI) and task type (STT vs. OET).

S3.7 Experimental Procedure

After providing informed consent, participants completed a pre-task questionnaire assessing demographic characteristics and prior AIGC-tool usage. Participants were assigned by the online experimental platform using computer-generated permuted-block randomization with a 1:1:1:1 allocation ratio, resulting in 80 participants in each of the four experimental conditions: SBI-STT, SBI-OET, TBI-STT, and TBI-OET.

Participants first viewed the instructional video corresponding to their assigned instructional condition. The SBI video lasted 6 min 28 s and the TBI video lasted 6 min 30 s. Immediately after the instructional manipulation, participants completed the two manipulation-check items.

Participants subsequently entered the image-generation stage and received either the structured-task or open-ended-task instructions according to their assigned condition. The image-generation stage lasted 10 min and was conducted under the standardized platform settings and restrictions described in Section S3.6.

At the end of the task, participants submitted the standardized task materials described in Section S3.5 and subsequently completed the measures of autonomy, competence, relatedness, and AI literacy self-awareness.

The full experimental session required approximately 20–25 min.

Supplementary Figure S3.3. Detailed experimental procedure.

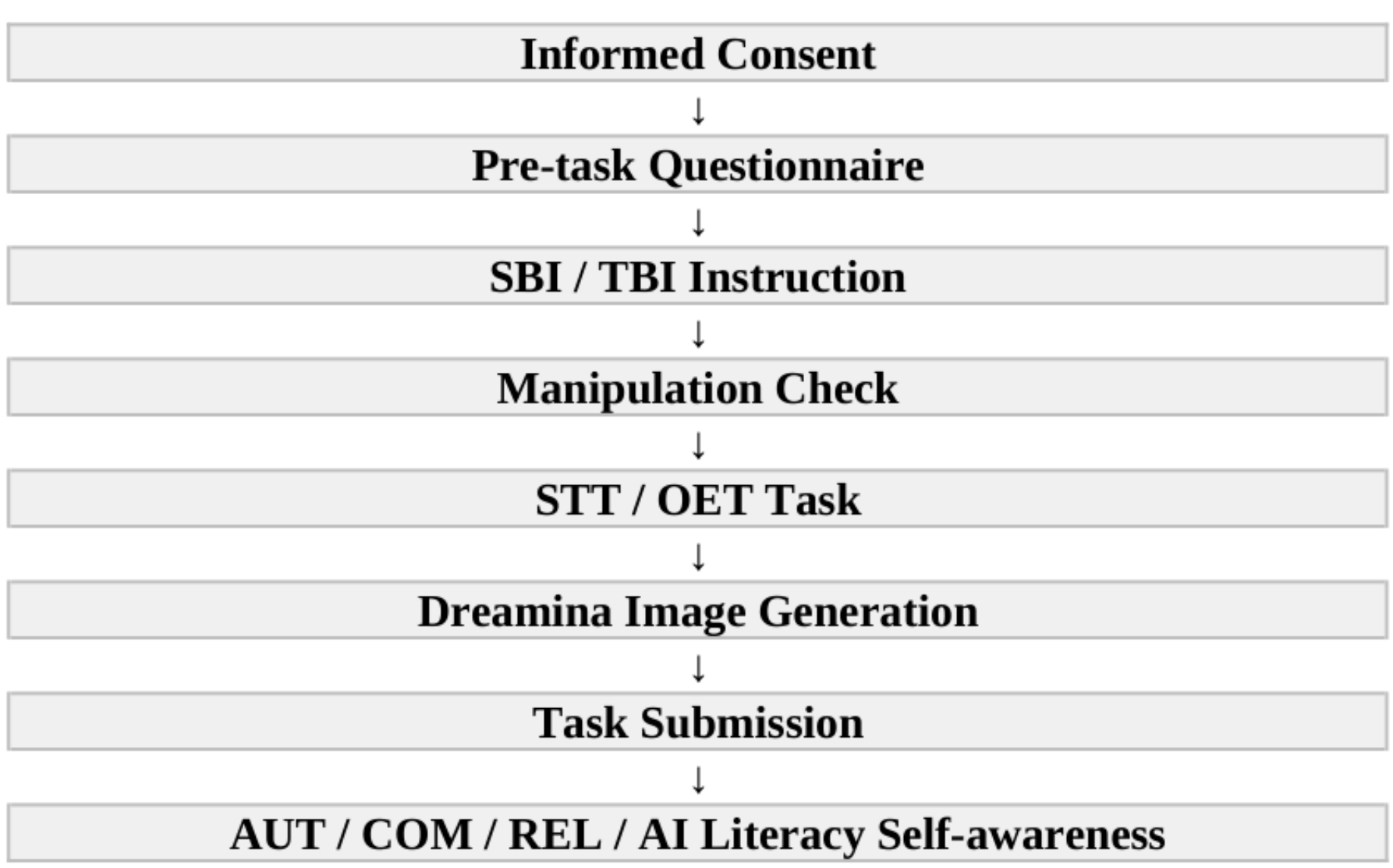

Supplement: Supplementary file 1 [file Supplementary_file_1.pdf]
